# Effectiveness and safety of segmentectomy vs. wedge resection for the treatment of patients with operable non‑small cell lung cancer: A meta‑analysis and systematic review

**DOI:** 10.3892/ol.2024.14469

**Published:** 2024-05-24

**Authors:** Jiawei Xiu, Shiqi Wang, Xilong Wang, Wei Xu, Yuhang Hu, Yujuan Hua, Shiguang Xu

**Affiliations:** 1 Department of Thoracic Surgery, General Hospital of Northern Theater Command, Shenyang, Liaoning 110016, P.R. China; 2 Graduate School, China Medical University, Shenyang, Liaoning 110122, P.R. China; 3 Department of Anaesthesiology, General Hospital of Northern Theater, Shenyang, Liaoning 110016, P.R. China

**Keywords:** non-small cell lung cancer, segmentectomy, operable, wedge resection, meta-analysis

## Abstract

The present study compared the differences in effectiveness and safety between segmentectomy (ST) and wedge resection (WR) in patients with operable non-small cell lung cancer (NSCLC). The PubMed, EMBASE, Cochrane Library and Web of Science databases were searched for papers published from inception until July 2023. The inclusion criteria were based on the population, intervention, comparator, outcomes and study designs. ROBINS-I was selected to assess the risk of bias and quality of evidence in the included non-randomised studies. Appropriate effect sizes were selected, and subgroup analyses, heterogeneity tests, sensitivity analyses and publication bias were applied. A total of 18 retrospective studies were included, involving 19,381 patients with operable NSCLC. The 5-year overall survival rate [hazard ratio (HR), 0.19; 95% confidence interval (CI), 0.04, 0.34; P=0.014; I^2^=76.3%], lung cancer-specific survival rate (HR, 0.3; 95% CI, 0.21, 0.38; P<0.01; I^2^=13.8%) and metastasis rate [odds ratio (OR), 1.56; 95% CI, 1.03, 2.38; P=0.037] in patients with operable NSCLC treated with WR were worse than those in patients treated with ST. The incidence of postoperative complications (OR, 0.44; 95% CI, 0.23, 0.82) in the WR group was lower than in the ST treatment group. There was no difference in postoperative recurrence (OR, 2.15; 95% CI, 0.97, 4.74; P=0.058) and mortality (risk difference, 0.04; 95% CI, −0.03, 0.11; P=0.287) between groups. Based on current evidence, patients with NSCLC treated with ST surgery have better postoperative survival but more complications than those patients treated with WT, while the effect of WR and ST on the recurrence rate and distant metastasis rate remains controversial.

## Introduction

Lung cancer is the 2nd most commonly diagnosed cancer after female breast cancer and the leading cause of cancer death worldwide. In 2020, there were 2.2 million new lung cancer cases and an estimated 1.8 million (18%) lung cancer-associated deaths globally (1). Surgery is the preferred treatment for early-stage lung cancer. With the development of modern techniques such as computed tomography screening and other imaging techniques, the early detection of smaller lesions has improved, increasing the number of surgeries (2). Lobectomy is usually recommended for patients with stage I non-small cell lung cancer (NSCLC) (3,4). Studies also confirmed that lobectomy combined with mediastinal lymph node dissection has a 5-year survival rate of ~60% (4,5). Considering that most patients with NSCLC are older, with a median age range diagnosis of 67.7–70 years (6,7), removing excess healthy tissue can seriously affect the patient's quality of life (8). Thus, alternative methods are recommended for these patients.

For those patients with severe comorbidities who cannot undergo lobectomy, segmentectomy (ST) and sublobectomy are currently being considered (9). In recent years, sublobectomy [ST or wedge resection (WR)] has gained attention since it can preserve lung function. WR is a nonanatomic procedure that removes the cancerous lung tissue surrounded by a margin of normal lung parenchyma. Its operation time is shorter because, during this procedure, the pulmonary vessels and bronchus do not need to be identified (10,11). By contrast, ST is an anatomic excision that requires the surgeon to carefully identify the location of the pulmonary blood vessels and bronchi. Due to the need for careful dissection and evaluation of intraparenchymal and hilar lymph nodes, ST is more technically demanding than WR (12). At present, WR is more commonly used than ST, accounting for ~80% of sublobectomy (13), but WR is generally considered less effective than anatomic ST for the following two reasons: i) In WR, lymph nodes in the tumor area are usually not removed immediately; and ii) the staple line edge of WR is closer to the tumor than that of ST (10). Various studies have produced inconsistent results regarding the impact of two surgical approaches i.e., WR and ST, on overall survival (OS) and lung cancer-specific survival (LCSS) in patients with operable NSCLC. Multiple studies have found no notable difference in OS or relapse-free survival between patients treated with ST and WR for NSCLC with ground glass opacity (GGO) as the primary clinical stage (14–17). Moreover, a meta-analysis of nine studies published in 2016 suggested that ST is associated with a higher OS rate than WR for patients with stage I NSCLC; however, no difference was observed for patients with stage IA NSCLC and tumors >2 cm in diameter (10). Another meta-analysis of 19 studies published in 2019 concluded that OS, LCSS and disease-free survival (DFS) after ST were markedly higher than those after WT in patients with NSCLC (18). In addition, recent clinical studies have shown that WR and ST are equally effective in NSCLC with a GGO diameter of 2–3 cm, with fewer complications, faster recovery of lung function and greater prevention of postoperative death from causes other than NSCLC (19,20). Still, this meta-analysis (18) only evaluated the difference between WR and ST in survival outcomes, and the latest studies (19,20) pointed out that WR may have clinically significant advantages in terms of postoperative complications, and recurrence and metastasis rates.

In the present study, a meta-analysis was performed to compare the outcomes of OS, LCSS, mortality, complication rate, recurrence and metastasis rates in patients with operable NSCLC receiving WR compared with those undergoing ST. These data further contribute to our understanding of surgical options for patients with NSCLC who received limited lung resection and provide more specific recommendations for clinical decision-making.

## Materials and methods

### Literature search

The current meta-analysis was performed according to the 2020 Preferred Reporting Items for Systematic Reviews and Meta-Analyses guidelines (21). The relevant articles were searched based on the population, intervention, comparison, outcomes and study principle (22). PubMed (https://pubmed.ncbi.nlm.nih.gov/), Embase (https://www.embase.com/), Web of Science (https://webofscience.com) and Cochrane Library (https://www.cochranelibrary.com/) were systematically searched for potentially eligible studies published from inception to July 2023, using the Medical Subject Headings terms ‘carcinoma, non-small-cell lung’, ‘pneumonectomy’, ‘sublobar resection’, ‘segmentectomy’ and ‘wedge resection’ and relevant keywords (Table SI). The literature retrieval and selection process were performed independently by two investigators and results were compared once the process was complete. Any discrepancy was solved by discussion.

### Eligibility criteria

The inclusion criteria were: i) Studies reporting on patients with operable NSCLC; ii) the intervention group received WR, while a control group received ST; iii) study endpoints included OS, LCSS, mortality, recurrence, metastasis and postoperative complications; iv) no restrictions on study design; and v) full text available. The exclusion criteria were: i) Reviews, case reports, dissertations, conference papers, chapters in handbooks and editorials; ii) studies not published in an international peer-reviewed journal; and iii) duplicate published studies.

### Data extraction

Two independent investigators performed data extraction. Any disagreements were resolved by a third reviewer. Data included the authors' name, publication year, study design and location, sample size, data sources, male proportion, mean age, stage of NSCLC, tumor size and study outcomes. The primary outcome was the 3- or 5-year OS, LCSS, mortality and postoperative complications; secondary outcomes included the recurrence and metastasis rates.

### Quality assessment

Due to the particularity of intervention methods, complete double-blind, randomized controlled studies (RCTs) are challenging. ROBINS-I was selected to assess the risk of bias and quality of evidence in the included non-randomised studies of interventions (23). Quality assessment was performed in duplicate by investigators separately.

The contents of the study evaluation included the following: Bias due to confounding, bias in the selection of participants into the study, bias in classification of interventions, bias due to deviations from intended interventions, bias due to missing data, bias in the measurement of outcomes and bias in the selection of the reported result. The categories for risk of bias judgments were: ‘Low risk’, ‘Moderate risk, ‘Serious risk’ and ‘Critical risk’ of bias.

### Statistical analysis

All analyses were performed using the Stata (version 15.1 SE; http://www.stata.com/stata15/). Odds ratios (ORs), hazard ratios (HRs), risk difference (RDs) and their corresponding 95% confidence intervals (Cis) were used to compare the outcomes. Studies providing 5-year OS numbers and 5-year OS HRs were separated for pooled analysis. For studies that reported effective sizes by subgroup, the subgroups were combined and the effect size for the entire sample was calculated. Statistical heterogeneity among the included studies was calculated using Cochran's Q-test, and the I2 index (I2>50% and Q-test P>0.10 indicated high heterogeneity). Given the clinical heterogeneity of the original studies (e.g., geographic, ethnic and others), the random effects model was used. P<0.05 was considered to indicate a statistically significant difference. A sensitivity analysis with a leave-one-out method was performed to assess the potential confounding effects and the robustness of the pooled results. If the pooled results after the exclusion of a study were inconsistent with the original pooled ones, the study was excluded as a potential confounder. Subgroup analysis was conducted to examine the survival outcome complications, and the recurrence and metastasis rates between WR and ST in different data sources [Surveillance, Epidemiology, and End Results (SEER) database or hospital], and tumor size (<1, 1–2, 2–3 and <3 cm). Publication bias was identified through the funnel plot, Egger regression and Begg tests.

## Results

### Study selection and characteristics

**Figure 1.**
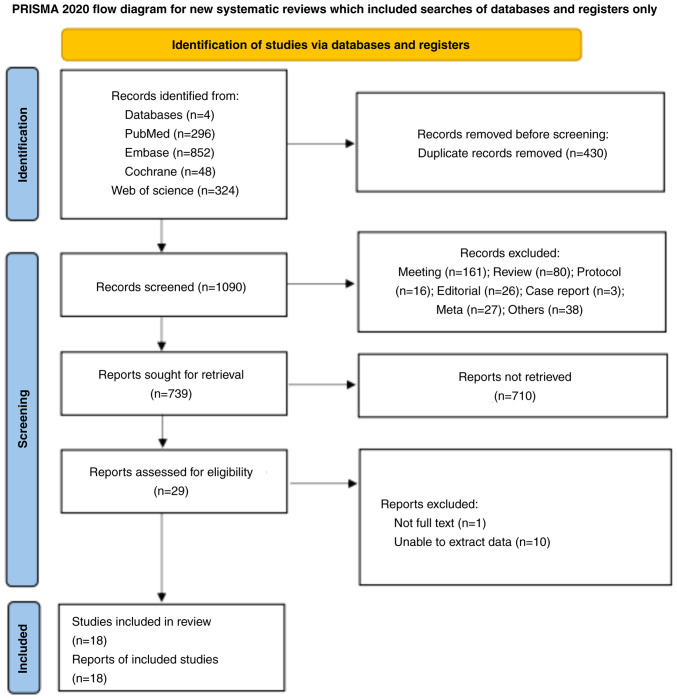
Flowchart of the study selection.

Fig. 1 presents the study selection process. The initial search resulted in 1,520 records. After removing 430 duplicates by automation tools and 1,062 records after reviewing the title and abstract, 29 studies were included. One study without a full text and 10 studies without interest outcomes were further excluded. Finally, 18 studies (19,20,24–39) were included in the current meta-analysis.

Table I shows the characteristics of the included studies. A total of 19,381 patients with operable NSCLC were included in the current meta-analysis; 14,611 in the WR group and 4,770 in the ST group. Studies were conducted in four countries, including America (n=2), China (n=10), Germany (n=1) and Japan (n=5). All of the included studies were retrospective. Nine studies had data from the SEER database, and the other nine obtained oncology data from hospitals. The mean age range of the included patients with operable NSCLC was 55.96–79.9 years. The range of female percentage was 30.3–79.09%. All of the patients with NSCLC included in the 12 studies that provided pathological staging of the tumor were evaluated as stage IA lung cancer. The mean tumor size range was 1.5–4 cm.

### Study quality

Table II shows the risk of bias assessment results of the included studies. Only three included studies had a moderate risk of bias, while the remaining 15 studies had a low risk. The main consideration of the three studies with ‘moderate risk’ was a bias due to potential confounding factors.

### OS

Data on the 3-(24,35) and 5-year OS (19,24,27,29,32, 35,37,38), and 5-year OS HRs (20,25,26,33,35,36,39) were extracted from the original studies to compare the postoperative OS of patients with operable NSCLC who received WR or ST. For patients with operable NSCLC, there was no significant difference in the 3- and 5-year OS numbers after WR and ST; yet, the 5-year OS rate after ST was higher than that in the WR group.

**Figure 2.**
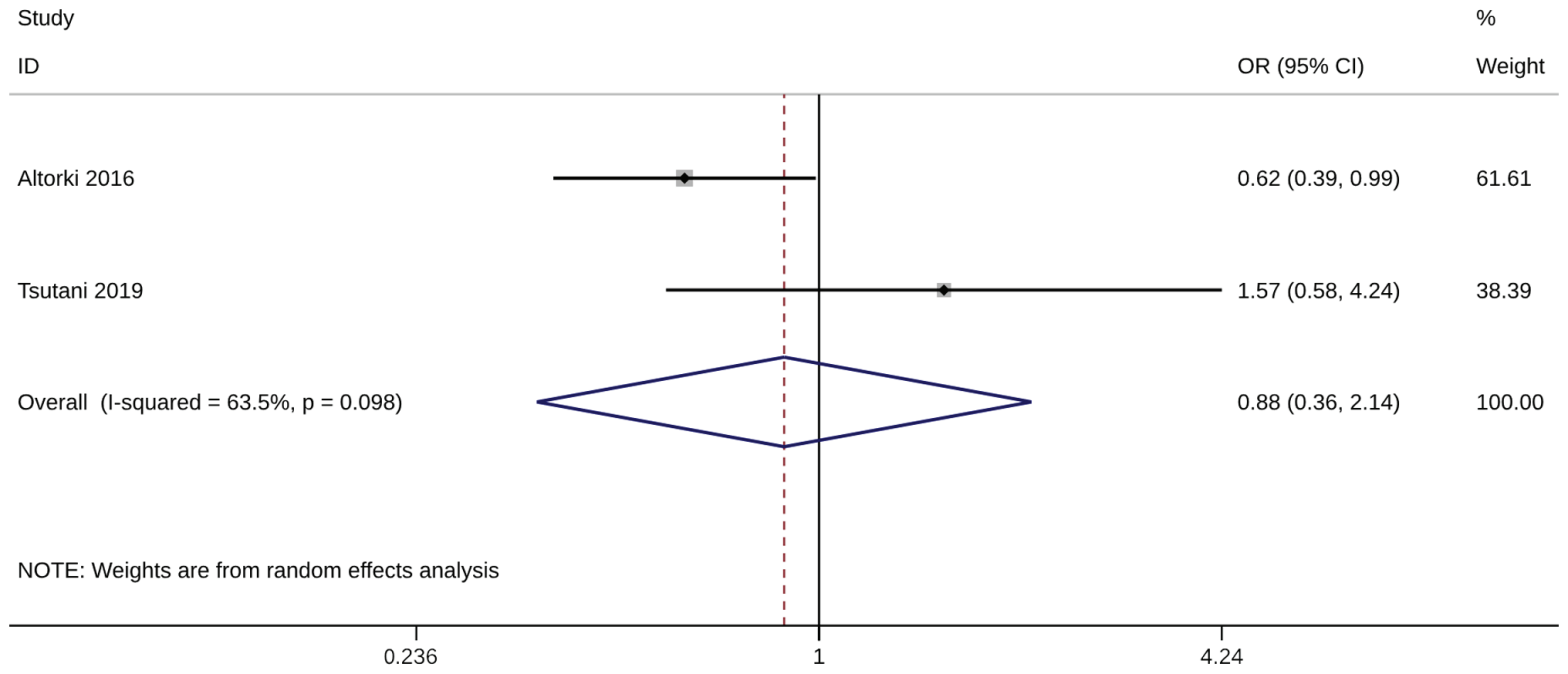
Forest plot of 3-year overall survival. OR, odds ratio; CI, confidence interval.

**Figure 3.**
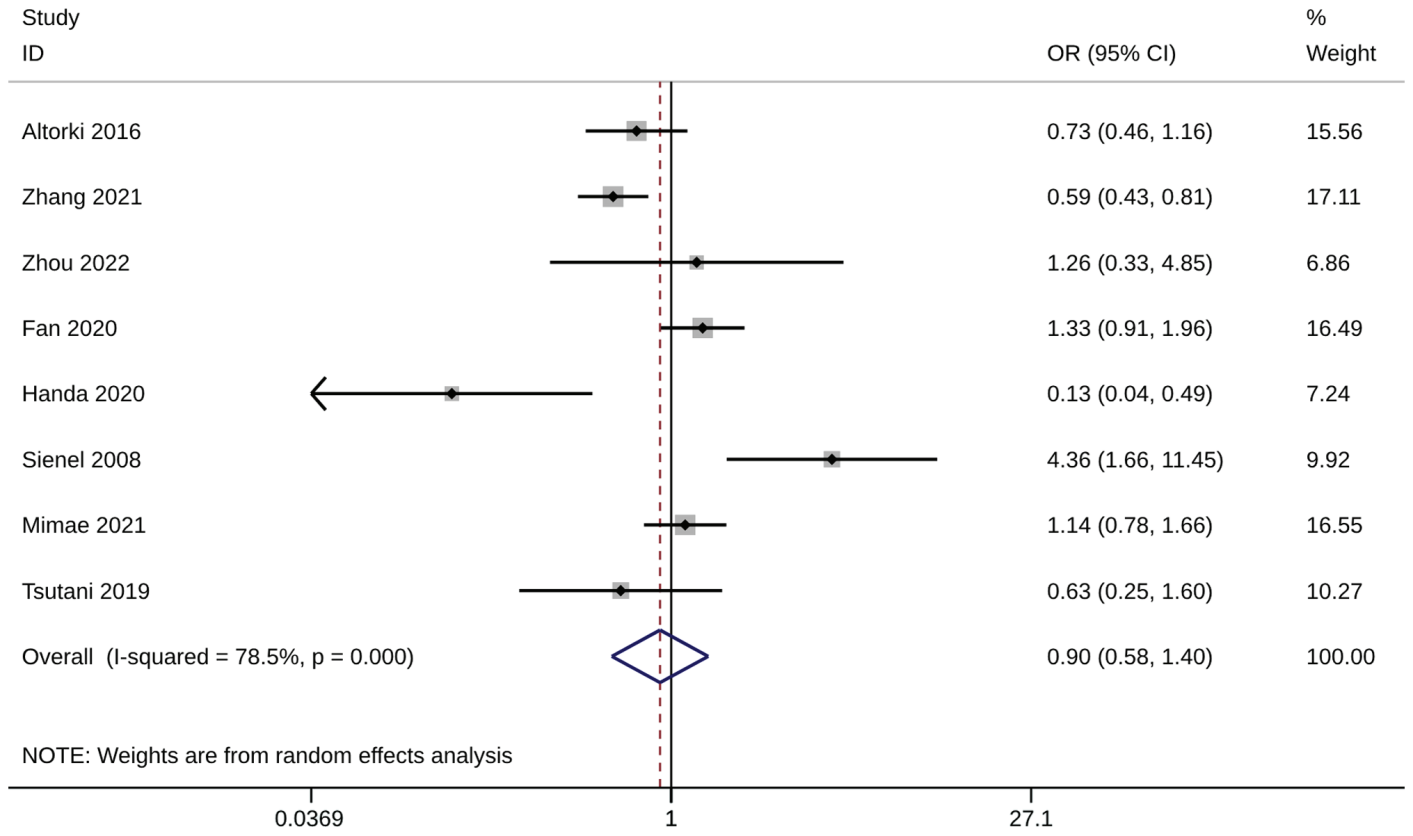
Forest plot of numbers of 5-year overall survival. OR, odds ratio; CI, confidence interval.

Several studies have examined the 3-year (n=2) (24,35) and 5-year (n=8) (19,24,27,29,32,35,37,38) OS numbers after WR or ST. The pooled analysis was only conducted when at least two adequately powered studies were available, and no significant difference in both survival numbers was observed between the WR and the ST group [(3-year OS, OR=0.88; 95% CI, 0.36, 2.14; P=0.782, I^2^=63.5%) and (5-year OS numbers: OR=0.90; 95% CI, 0.58, 1.40; P=0.649, I^2^=78.5%; Figs. 2 and 3)]. The sensitivity analysis results indicated that the meta-analysis findings were robust, with consistent results remaining unchanged even when excluding any single study (Fig. S1).

**Figure 4.**
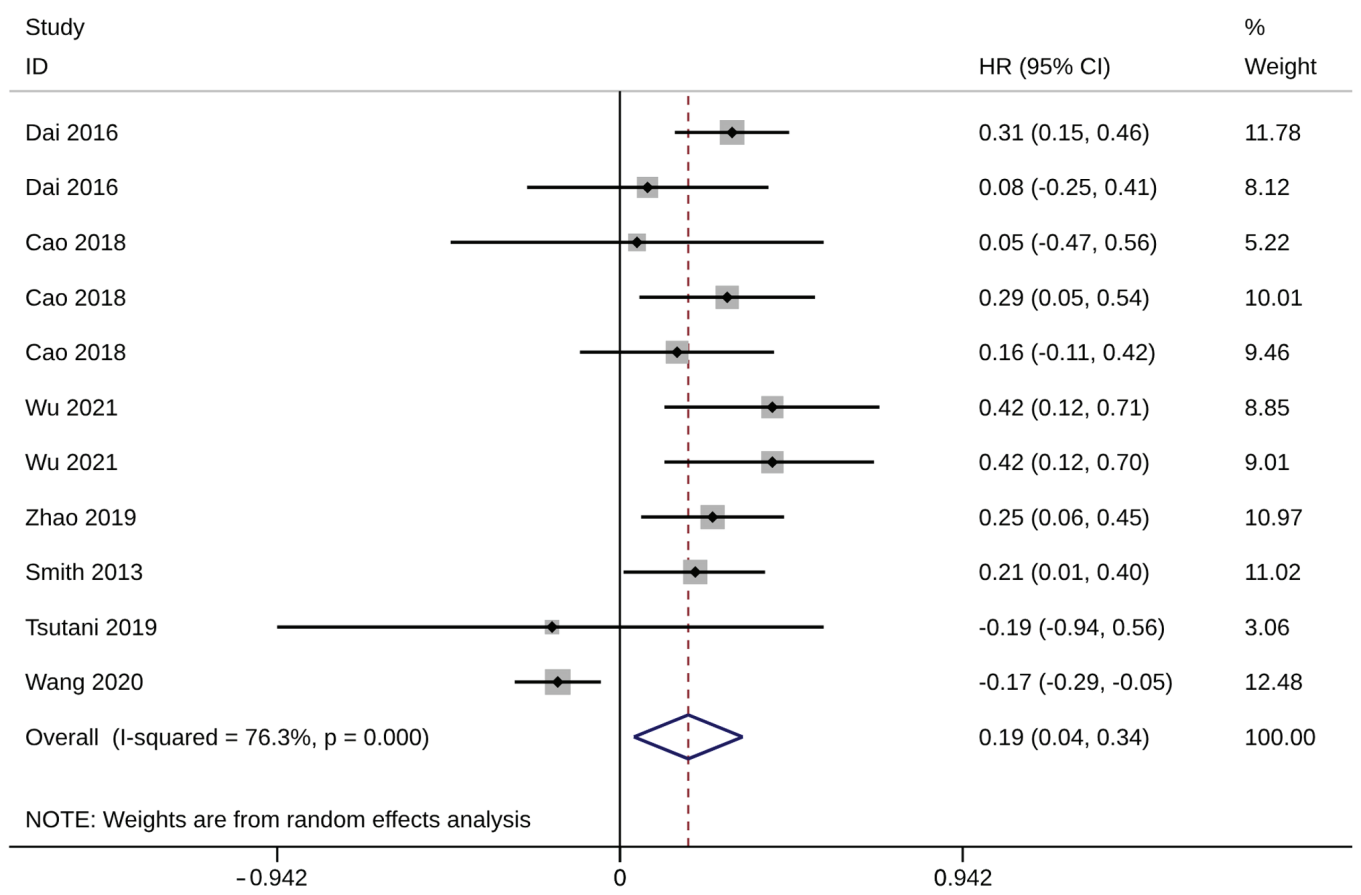
Forest plot of HR of 5-year overall survival. HR, hazard ratio; CI, confidence interval.

Seven studies with 11 trials (20,25,26,33,35,36,39), including 15,413 patients with operable NSCLC, reported on the HRs of 5-year OS. All these studies were included in the quantitative synthesis, and meta-analysis showed that with significant heterogeneity, 5-year OS was significantly higher after ST than after WR (HR, 0.19; 95% CI, 0.04, 0.34; P=0.014; I^2^=76.3%; Fig. 4). The sensitivity analysis results showed that the meta-analysis results were robust, and the advantage of ST remained unchanged when any one study was excluded (Fig. S2).

### LCSS

**Figure 5.**
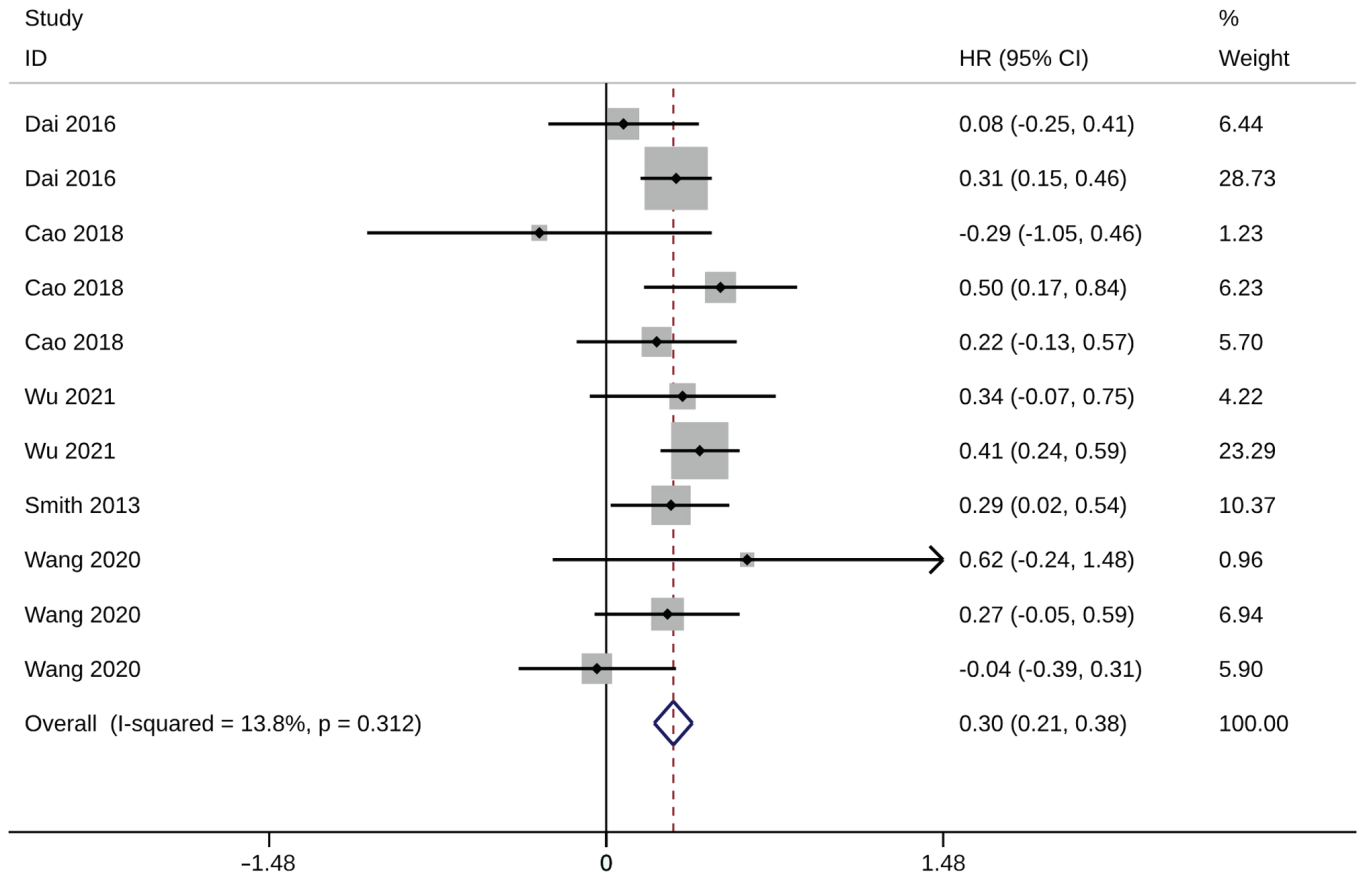
Forest plot of lung cancer-specific survival. HR, hazard ratio; CI, confidence interval.

Five studies with 11 trials reported on LCSS after operation. HR was used to compare LCSS between groups. Compared with the ST controls, significantly higher LCSS was observed in ST groups compared with WR groups (HR, 0.30; 95% CI, 0.21, 0.38; P<0.01). Heterogeneity was insignificant, so the fixed-effects model was used for the analysis (I^2^=13.8%; Fig. 5). Sensitivity analysis demonstrated the robustness of meta-analysis results since the advantage of ST remained unchanged after excluding any of these studies (Fig. S3).

### Mortality

**Figure 6.**
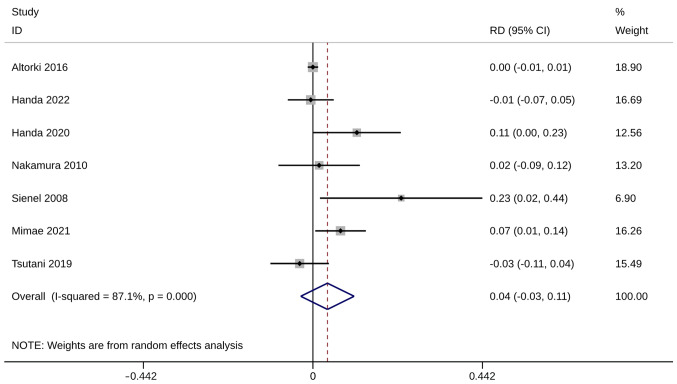
Forest plot of mortality. CI, confidence interval; RD, risk difference.

Mortality was evaluated using six studies with seven trials. No significant difference in mortality was observed between ST and WR groups (RD, 0.04; 95% CI, −0.03, 0.11; P=0.287). Heterogeneity was significant so the random-effects model was used for analysis (I^2^=87.1%; Fig. 6). The sensitivity analysis results were robust (Fig. S4).

### Complications

**Figure 7.**
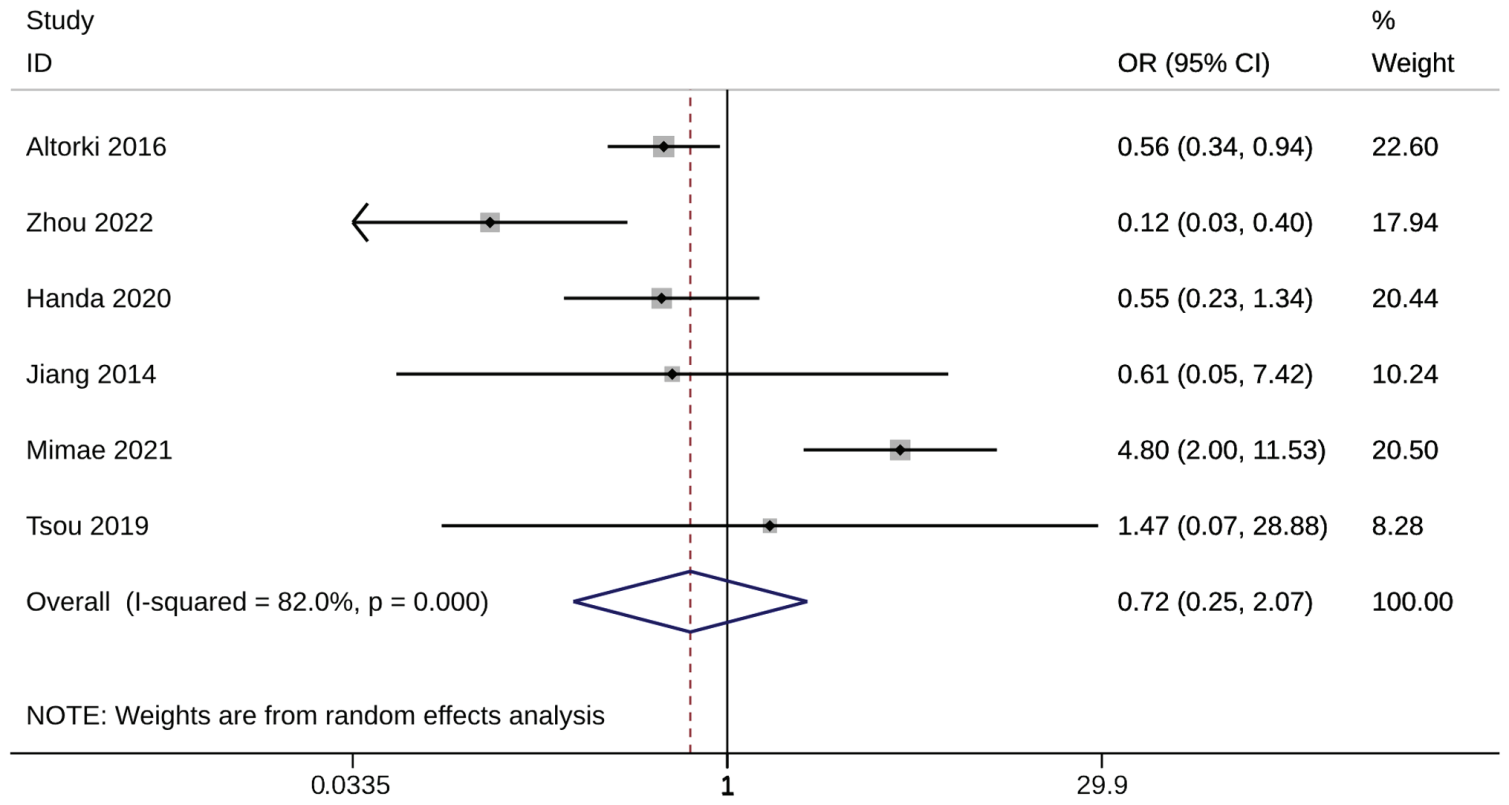
Forest plot of complication. OR, odds ratio; CI, confidence interval.

Mortality was evaluated using six studies. There was no significant RD in complication incidence between ST and WR groups (OR, 0.72; 95% CI, 0.25, 2.07; P=0.536). Heterogeneity was significant so the random-effects model was used for analysis (I^2^=82%; Fig. 7). The sensitivity analysis showed that when Mimae et al (38) was excluded, the pooled results were inconsistent with the original meta-analysis results (Fig. S5). Mimae et al (38) may be the source of heterogeneity, and meta-analysis results after exclusion showed that the complication rate in the WR group was significantly lower than that in the ST group (OR, 0.44; 95% CI, 0.23, 0.82; Table SI, Table SII, Table SIII).

### Recurrence rate

**Figure 8.**
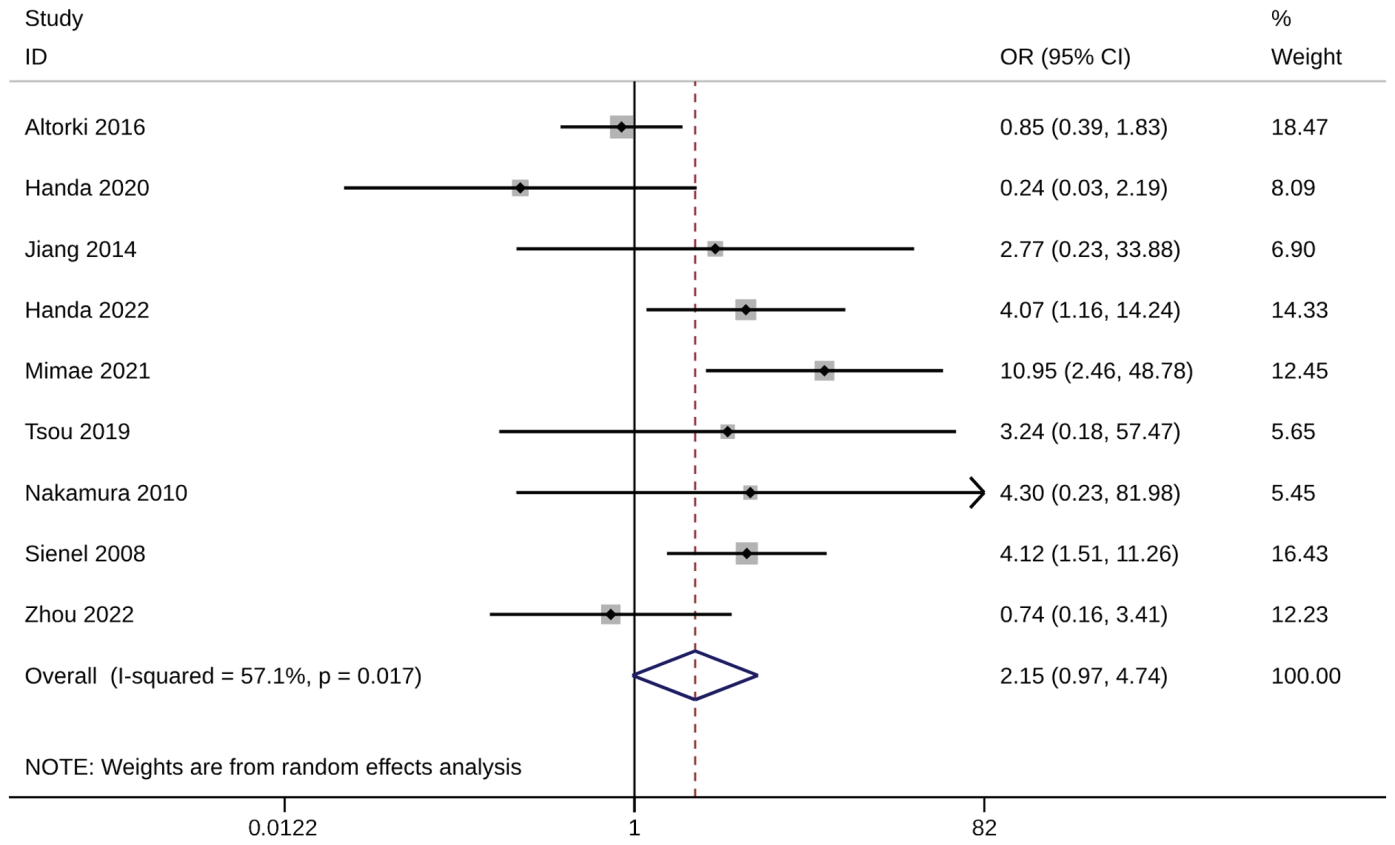
Forest plot of recurrence rate. OR, odds ratio; CI, confidence interval.

There were nine of the 18 included studies that compared recurrence rates between groups. No difference in recurrence rate was observed between ST and WR groups (OR, 2.15; 95% CI, 0.97, 4.74; P=0.058). Heterogeneity was significant so the random-effects model was used for analysis (I^2^=57.1%; Fig. 8). When the studies of Altorki et al (24), Handa et al (29) and Zhou et al (37) were excluded, the pooled results were inconsistent with the original meta-analysis results (Fig. S6). The aforementioned three included studies may be the source of heterogeneity, and meta-analysis results after exclusion showed that the recurrence rate in the WR group was significantly higher than in the ST group, with OR >1 and 95% CI not included 1 (Table SIII).

### Metastasis rate

**Figure 9.**
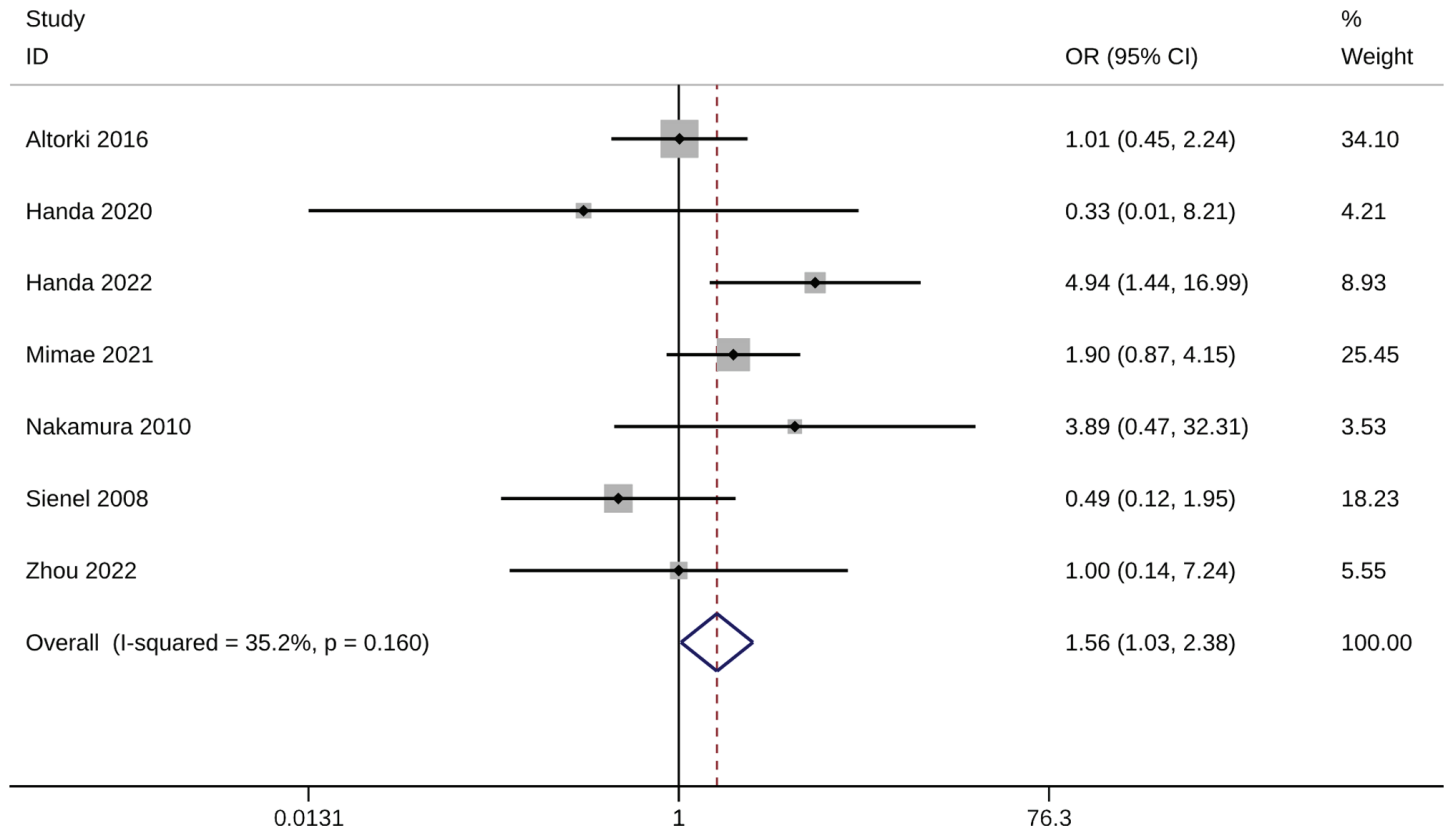
Forest plot of metastasis rate. OR, odds ratio; CI, confidence interval.

The metastasis rate was evaluated using six studies with seven trials. A significantly higher metastasis rate was observed in WR groups than in ST groups (OR, 1.56; 95% CI, 1.03, 2.38; P=0.037). Heterogeneity was not significant, so the fixed-effects model was used for analysis (I^2^=35.2%; Fig. 9). The sensitivity analysis results were robust (Fig. S7).

### Data sources subgroup-analysis

Given the heterogeneity of NSCLC data sources, subgroup analysis divided studies into SEER database and hospital groups. Considering the number of studies, all subgroup analyses were performed using six outcome variables, excluding 3-year OS numbers and LCSS.

In the SEER database subgroups, the 5-year OS rate in the ST group was higher than that in the WR group (HR, 0.20; 95% CI, 0.05, 0.35; P=0.01; I^2^=78.3%; n=10; Fig. S8), and the complication rate in the WR group was lower than that in the ST group (OR, 0.56; 95% CI, 0.34, 0.94; P=0.027; n=1; Fig. S9). No difference was observed in other outcomes (Figs. S10 and 11).

In hospital subgroups, higher recurrence (OR, 2.71; 95% CI, 1.22, 6.02; P=0.015; n=8) and metastasis rate (OR, 1.85; 95% CI, 1.12, 3.05; P=0.016; n=6) were observed in WR subgroups compared with ST groups (Figs. S12 and 13).

### Tumor size subgroup-analysis

Stratified by the average tumour size of enrolled patients with NSCLC, the study was divided into <1, 1–2, 2–3 and <3 cm, and not provided groups. Tumour size subgroup analyses were only performed for 5-year OS rate and LCSS.

For patients with NSCLC with tumor size <1 cm, LCSS in the ST group was superior to WR (HR, 0.3; 95% CI, 0.16, 0.44; P<0.01; n=4), while there was no difference in 5-year OS rate (HR, 0.22; 95% CI, −0.03, 0.47; P=0.089; n=3). When the tumor size was 1–2 cm, the 5-year OS rate (HR, 0.32; 95% CI, 0.20, 0.44; P<0.01; n=3; Fig. S14) and LCSS (OR, 0.35; 95% CI, 0.22, 0.48; P<0.01; n=4; Fig. S15) in the ST group were better than those in the WR group. In patients with NSCLC with tumor size <3 cm, the 5-year OS rate in the WR group was higher (HR, −0.17; 95% CI, −0.29, −0.05; P=0.005; n=1) and the LCSS was lower than that in the ST group (HR, 0.29; 95% CI, 0.02, 0.55; P=0.032; n=1). There was no difference in OS rate and LCSS 5 years after WR or ST in patients with tumor size 2–3 cm (Figs. S14 and 15).

### Publication bias and sensitivity analysis

Publication bias was assessed using a funnel plot, Egger regression test, Begg test and trim and fill method. According to Fig. S16, Fig. S17, Fig. S18, Fig. S19, Fig. S20, Fig. S21, Fig. S22, the Egger regression test and the Begg test results, no publication bias was observed in all primary or secondary outcomes (Table SII).

The results of the sensitivity analysis found that Mimae et al (38) may be the source of heterogeneity in complication rate (Fig. S5), and Altorki et al (24), Handa et al (28), or Zhou et al (37) were identified as the potential confounders of recurrence rate (Fig. S6).

## Discussion

A sublobar resection, which includes ST and WR, is considered a possible alternative to lobectomy (33), especially for patients with stage I NSCLC (40). Yet, specific target populations and postoperative prognostic outcomes of ST and WR remain controversial (32).

After excluding possible confounders, the findings of the present study suggest that 5-year OS, LCSS and metastasis rates in patients with operable NSCLC treated with WR are worse than those in patients treated with ST. However, the incidence of postoperative complications in WR patients was significantly lower than in the ST treatment group. Based on current evidence, ST may be the best option for patients with NSCLC whose tumor size range of 1–2 cm in terms of postoperative OS and LCSS. Clinical data from hospitals can better support the advantages of ST surgical methods in recurrence and metastasis rates. Also, the results related to postoperative survival in patients with NSCLC who received WR and ST were consistent with previous meta-analyses (10,12,18).

Numerous previous studies have compared the outcomes of patients with stage I NSCLC undergoing cuneiform and ST (10,18,41). The findings of the current study on OS and LCSS are consistent with those studies. Hou et al (10) analyzed 19 studies (3,604 patients receiving ST and 10,593 receiving WR) and concluded that survival and LCSS were higher for ST than for WR. Mata-analysis results published by Zhang et al (18) suggested that ST had markedly better OS, LCSS and DFS than WR in patients with stage I NSCLC who underwent lobectomy. Moreover, a Bayesian network meta-analysis published in 2022 showed that ST has marked benefits compared with WR for OS and DFS in patients with early-stage NSCLC (41). At the same time, compared with the aforementioned studies, the present meta-analysis included more recent research results, and the analysis results were more convincing. Previous studies have only included a limited number of original documents and these documents are quite old. The present study included 18 recent documents, which will make the findings more convincing. In addition, the previous studies are compared in the following paragraphs. By comparing a previously published study (29) and review (10), it was shown that recently published studies (29) report different results compared with older studies (10). For example, Hou et al (10) found comparable results between ST and WR with regards to OS when assessing studies published between 2005 and 2010, but OS was also higher for ST than WR when assessing studies published between 2010 and 2015, which may be due to the continuous improvement and development of ST surgical techniques. A study published in Handa in 2022 comparing the survival outcomes of complex ST and WR when lobectomy is not possible, lung cancer with a ‘complex site’ solid component size ≤2.0 cm may require complex ST instead of WR (29). This complex ST requires the surgeon to separate and segment the appropriate segmental bronchi, arteries and veins more peripherally and, in some cases, to cut into the lung parenchyma. Several intersegmental planes with safe surgical margins must be made. Compared with WR resection of lung tumors with normal parenchymal margins, the aforementioned procedure can effectively improve the postoperative survival of patients. Meanwhile, a more recent study has further findings on the size of the tumors suitable for the two surgeries. In their meta-analysis, Hou et al (10) suggested that the larger the tumor size for patients with stage I NSCLC, the greater the advantage of ST over WR. Both meta-analyses were published later, and the present study found no difference in the effect of ST and WR on survival outcomes in patients with NSCLC with tumor sizes >2 cm.

For some types of tumors, the recurrence rate depends primarily on the appropriateness of selecting the initial surgical resection area (42). There is an idea that patients who undergo limited resection, WR or ST, have a markedly increased risk of intrathoracic recurrence (43,44). Another view is that there is no notable difference in survival between patients with stage I NSCLC undergoing sublobectomy (cuneiform resection and ST) and standard lobectomy, especially for early NSCLC (45,46). NSCLC is a malignancy that affects mainly the elderly, and the removal of excess healthy tissue can seriously affect the patient's quality of life (6,8). The present meta-analysis aimed not to select the best surgical resection between WR and ST, but to clarify whether WR has the same survival, recurrence and metastasis rates as ST as WR preserves more normal tissue (14). The current results suggest that WR, which preserves more normal tissue, may not be prioritized when both resection methods are suitable for patients.

For patients with NSCLC, the choice of postoperative recurrence is more relevant than the choice of surgical resection margin. Sato et al (47) demonstrated that the accurate identification of adequate surgical margins plays a crucial role in preventing recurrence at the margins. Although no conclusion has been reached, it is hypothesised that 20-mm incision margin for a full lung and 15-mm incision margin for a depleted lung are the appropriate choices (48). Tsutani et al (49) compared cancer control between ST and WR in patients with clinical stage IA NSCLC and found that 36/195 (18.5%) patients undergoing WR and 14/262 (5.3%) patients undergoing ST experienced recurrence after surgery. ST has better tumor control than WR. Suzuki et al (50) reported that the median incision margin of pathological surgery was 15 mm, and the 5-year recurrence-free survival rate was 99.7%. Sublobectomy with adequate surgical margins provides adequate local control and recurrence-free survival for clinically resectable lung cancer. Zhou et al (37) showed that WR may be better suited for peripheral lesions, whereas ST is better suited for deeper lesions that cannot be accessed by WR.

Adequate lymph node dissection is the key to preventing metastasis and recurrence. For patients with early NSCLC ≥3 cm in diameter, evaluated lymph nodes in patients undergoing sublobectomy are associated with better OS and LCSS (51). Baig et al (52) reported that ST has a higher long-term survival rate than WR for second primary lung cancer after a prior lobectomy. WR for second primary lung cancer markedly improves OS when adequate lymph node dissection is performed.

The results of the study showed that the outcomes of WR in terms of survival, recurrence and metastasis were not favourable; however, a study by Handa et al (29) included demonstrated generally lower toxicity and a significantly lower incidence of postoperative complications in WR as compared to the ST group. Regarding short-term surgical outcomes such as surgical time, surgical blood loss and postoperative hospital stay, WR is less damaging than ST (29). Similar to previous findings, WR had 3.15 fewer complications than ST. Notably, there were no serious postoperative complications, and postoperative mortality was comparable to ST (29), which also provides surgical options for patients with early NSCLC who are older or have severe comorbidities.

The present study has some limitations. First, a large amount of retrospective data can bring uncertainty and concerns to a conclusion; thus, more prospective studies and RCTs are needed to make up for this shortcoming. Second, a great heterogeneity in the included studies may lead to some bias. Third, specific surgical methods for different ST and WR, such as complex ST and simple ST included in the studies, were not discussed. Also, due to limited original data, the current study did not conduct a subgroup analysis of patients of different ages, which could have further refined the target population of different surgical methods.

A meta-analysis, which was published in 2019, incorporated 15 studies, four of which were prospective (18). Although the number of studies included in the present study is lower than that of the study of Zhang et al (18), the time range of the literature search has been extended from before April 30, 2018 to July 2023. The 16 studies included in the Bayesian meta-analysis published in 2022 were all retrospective studies, similar to those in the current study (41). Therefore, in terms of the included studies, it is hypothesised that the present meta-analysis is just as compelling as previous meta-analyses. As a result, it contributes to the exploration of postoperative recurrence and metastasis of NSCLC, which is somewhat innovative.

The data of the current study suggest that the postoperative survival rate of patients with operable NSCLC after WT is not equivalent to that of ST; however, the postoperative complication rate of WT is lower than that of ST. The equivalence of recurrence rate and distant metastasis rate remains controversial. Practitioners should not blindly use WT instead of ST in clinical practice. More prospective studies and RCTs are needed to further analyse the differences in recurrence and metastasis rates between ST and WR surgical modalities.

## Supplementary Material

Supporting Data

Supporting Data

## Figures and Tables

**Table I.** Characteristics of the included studies.

First author(s), year	Country	Study design	Data sources	Sample size, n (WR/ST)	Age, years	Female, %	Stage of NSCLC	Mean tumor size, cm (WR/ST)	Outcomes	(Refs.)
Altorki et al, 2016	US	Retrospective study	SEER database	160/129	72.66	57.09	IA	1.5/1.7	OS, AEs, death	(24)
Dai et al, 2016	China	Retrospective study	SEER database	3,316/769	68.3	61.27	IA	<2	OS, LCSS	(26)
Cao et al, 2018	China	Retrospective study	SEER database	3,007/809	n/a	58.94	IA	<3	OS, LCSS	(25)
Wu et al, 2021	China	Retrospective study	SEER database	995/182	n/a	61.43	IA	<2	OS, LCSS	(36)
Zhang et al, 2021	China	Retrospective study	SEER database	736/194	79.9	60.54	IA	<4	OS	(19)
Zhao et al, 2019	China	Retrospective study	SEER database	686/686	69	63.37	IA	n/a	OS	(39)
Zhou et al, 2022	China	Retrospective study	Hospital	100/100	57.45	44.5	n/a	2-3	AEs, OS	(37)
Fan et al, 2020	China	Retrospective study	SEER database	851/175	n/a	64.33	IA	n/a	OS	(27)
Handa et al, 2022	Japan	Retrospective study	Hospital	179/179	69.5	50.55	IA	1.5	Death	(28)
Handa et al, 2021	Japan	Retrospective study	Hospital	61/61	67.5	42.62	IA	<2	AEs, death, OS	(29)
Jiang et al, 2014	China	Retrospective study	Hospital	15/19	64.2	64.71	n/a	<1	AEs	(30)
Nakamura et al, 2011	Japan	Retrospective study	Hospital	84/38	74.18	38.52	n/a	n/a	Death	(31)
Sienel et al, 2008	Germany	Retrospective study	Hospital	31/56	65.67	29.89	IA	n/a	Death, OS	(32)
Smith et al, 2013	US	Retrospective study	SEER database	1,568/378	70	55.76	IA	≤3	OS, LCSS	(33)
Mimae et al, 2021	Japan	Retrospective study	Hospital	191/279	n/a	n/a	n/a	n/a	Death, AEs, OS	(38)
Tsou et al, 2020	China	Retrospective study	Hospital	273/57	55.96	79.09	n/a	<1	AEs	(34)
Tsutani et al, 2019	Japan	Retrospective study	Hospital	60/39	76	30.3	n/a	n/a	Death, OS	(35)
Wang et al, 2020	China	Retrospective study	SEER database	2,298/620	76.7	56.5	IA	≤3	LCSS	(20)

WR, wedge resection; ST, segmentectomy; NSCLC, non-small cell lung cancer; OS, overall survival; AEs, adverse effects; LCSS, lung cancer-specific survival; n/a, not applicable.

**Table II.** Quality evaluation by ROBINS-I.

First author(s), year	D1	D2	D3	D4	D5	D6	D7	Overall	(Refs.)
Altorki et al, 2016	Low	Low	Low	Low	Low	Low	Low	Low	(24)
Wu et al, 2021	Moderate	Low	Low	Low	Low	Low	Low	Moderate	(36)
Dai et al, 2016	Low	Low	Low	Low	Low	Low	Low	Low	(26)
Cao et al, 2018	Low	Low	Low	Low	Low	Low	Low	Low	(25)
Zhang et al, 2021	Moderate	Low	Low	Low	Low	Low	Low	Moderate	(19)
Zhao et al, 2019	Low	Low	Low	Low	Low	Low	Low	Low	(39)
Zhou et al, 2022	Low	Low	Low	Low	Low	Low	Low	Low	(37)
Fan et al, 2020	Low	Low	Low	Low	Low	Low	Low	Low	(27)
Handa et al, 2022	Low	Low	Low	Low	Low	Low	Low	Low	(28)
Handa et al, 2021	Low	Low	Low	Low	Low	Low	Low	Low	(29)
Jiang et al, 2014	Low	Low	Low	Low	Low	Low	Low	Low	(30)
Nakamura et al, 2011	Moderate	Low	Low	Low	Low	Low	Low	Moderate	(31)
Sienel et al, 2008	Low	Low	Low	Low	Low	Low	Low	Low	(32)
Mimae et al, 2021	Low	Low	Low	Low	Low	Low	Low	Low	(38)
Tsou et al, 2020	Low	Low	Low	Low	Low	Low	Low	Low	(34)
Tsutani et al, 2019	Low	Low	Low	Low	Low	Low	Low	Low	(35)
Wang et al, 2020	Low	Low	Low	Low	Low	Low	Low	Low	(20)
Smith et al, 2013	Low	Low	Low	Low	Low	Low	Low	Low	(33)

D1, Bias due to confounding; D2, Bias in selection of participants into the study; D3, Bias in classification of interventions; D4, Bias due to deviations from intended interventions; D5, Bias due to missing data; D6, Bias in measurement of outcomes; D7, Bias in selection of the reported result.

## Data Availability

The data generated in the present study are included in the figures and/or tables of this article.
