# Supplementary material for: Effectiveness and safety of segmentectomy vs. wedge resection for the treatment of patients with operable non‑small cell lung cancer: A meta‑analysis and systematic review
Source: Oncol Lett. 2024 May 24;28(1):336. doi: 10.3892/ol.2024.14469 (PMC11153982; doi:10.3892/ol.2024.14469)

Figure S1. Sensitivity analysis of numbers of 5-year overall survival. CI, confidence interval.

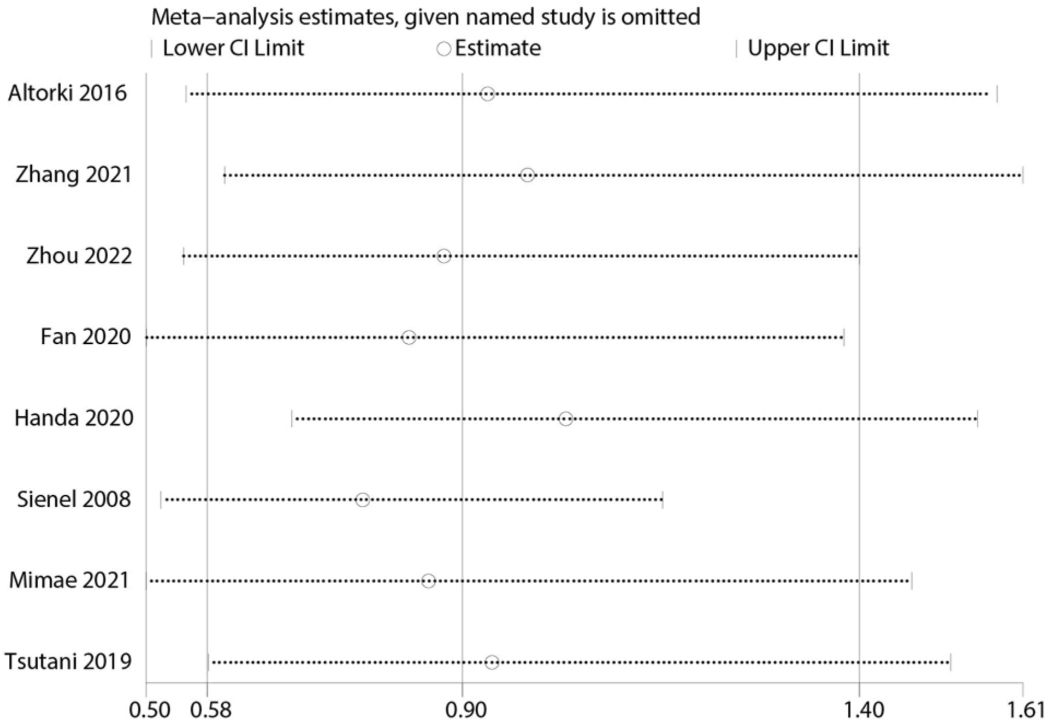

Figure S2. Sensitivity analysis of HR of the 5-year overall survival. CI, confidence interval.

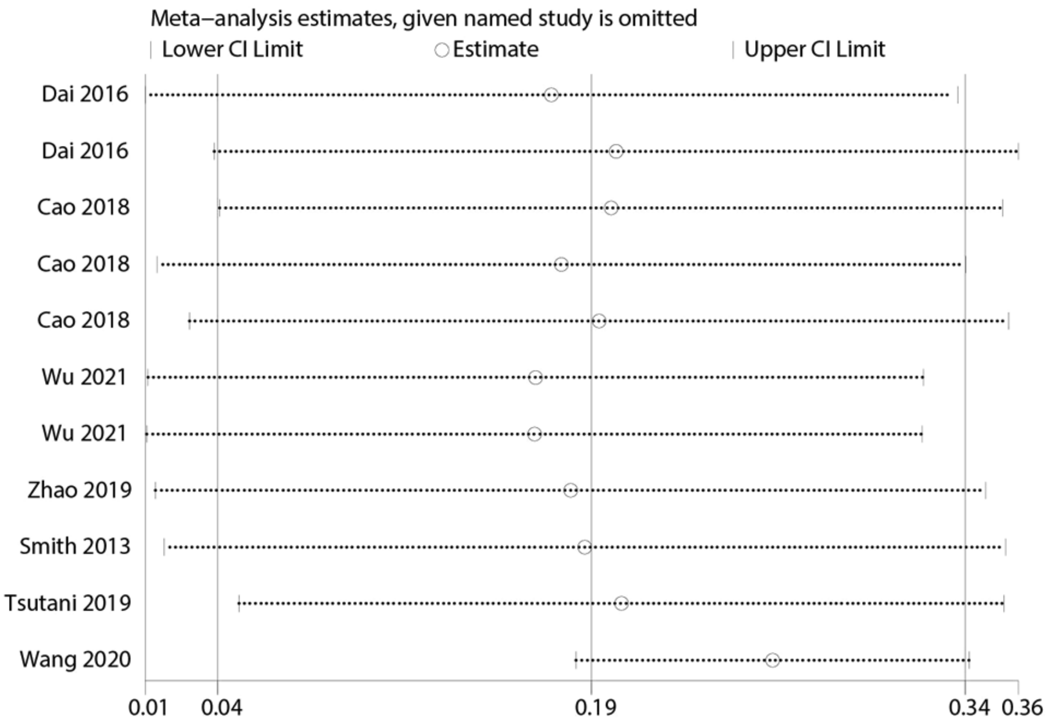

Figure S3. Sensitivity analysis of lung cancer-specific survival. CI, confidence interval.

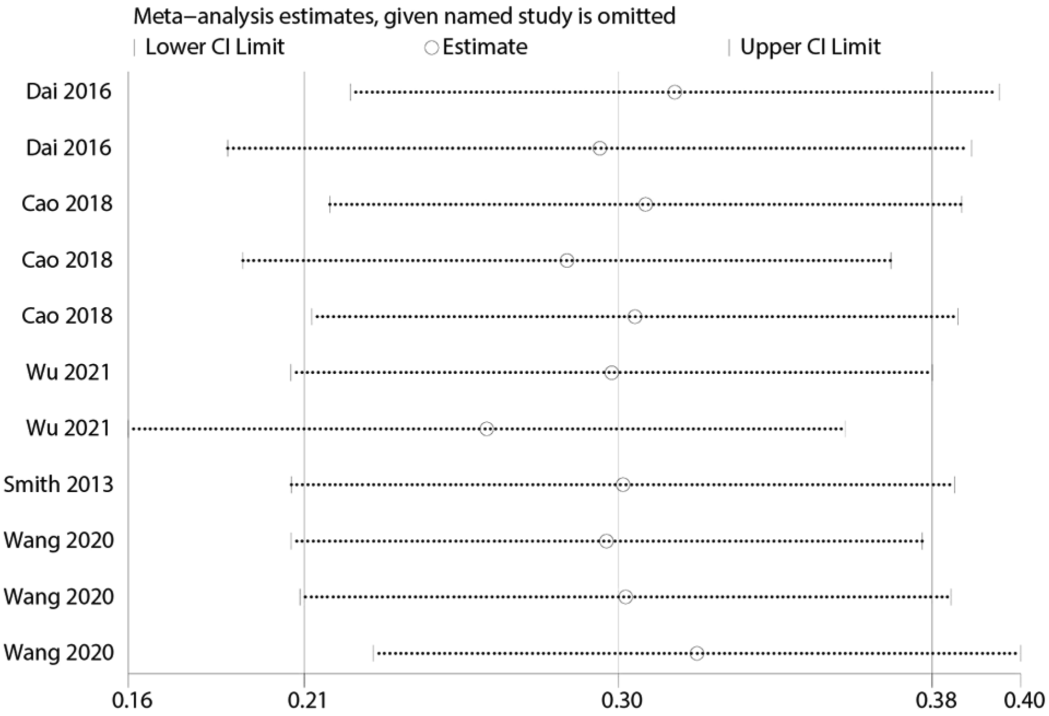

Figure S4. Sensitivity analysis of mortality. CI, confidence interval.

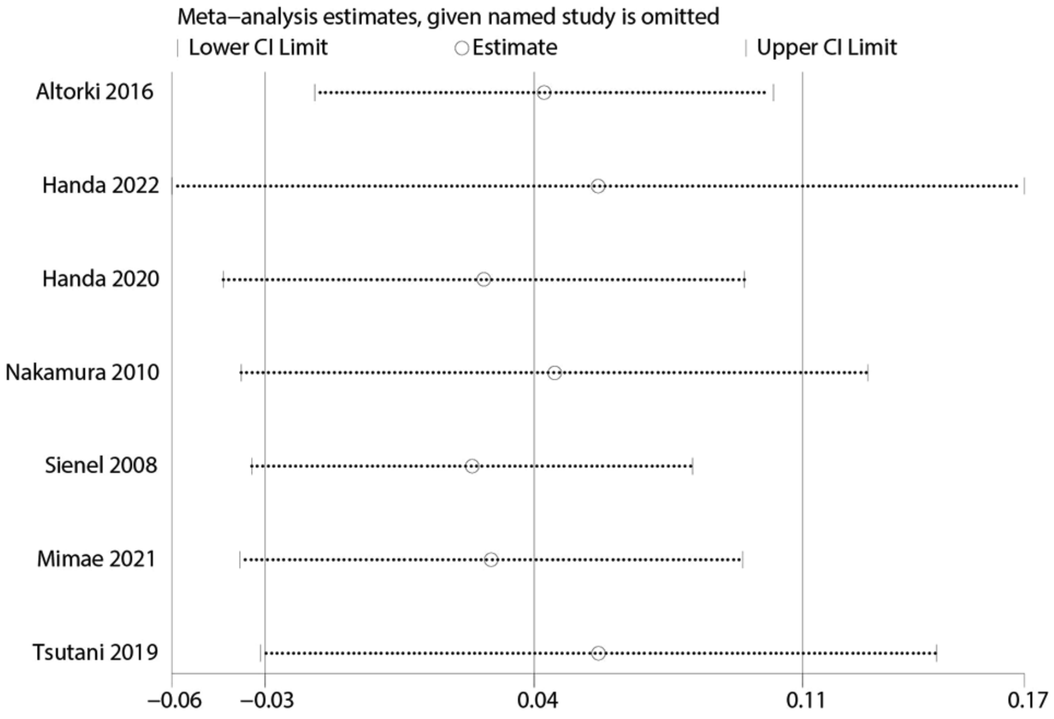

Figure S5. Sensitivity analysis of complication. CI, confidence interval.

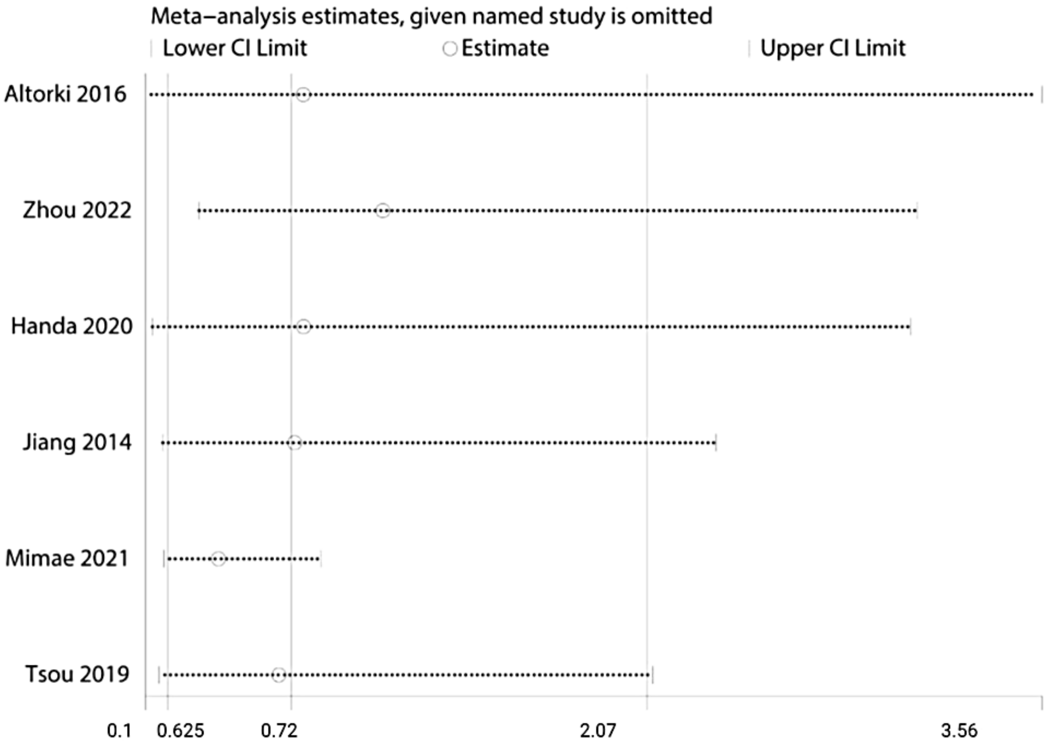

Figure S6. Sensitivity analysis of recurrence rate. CI, confidence interval.

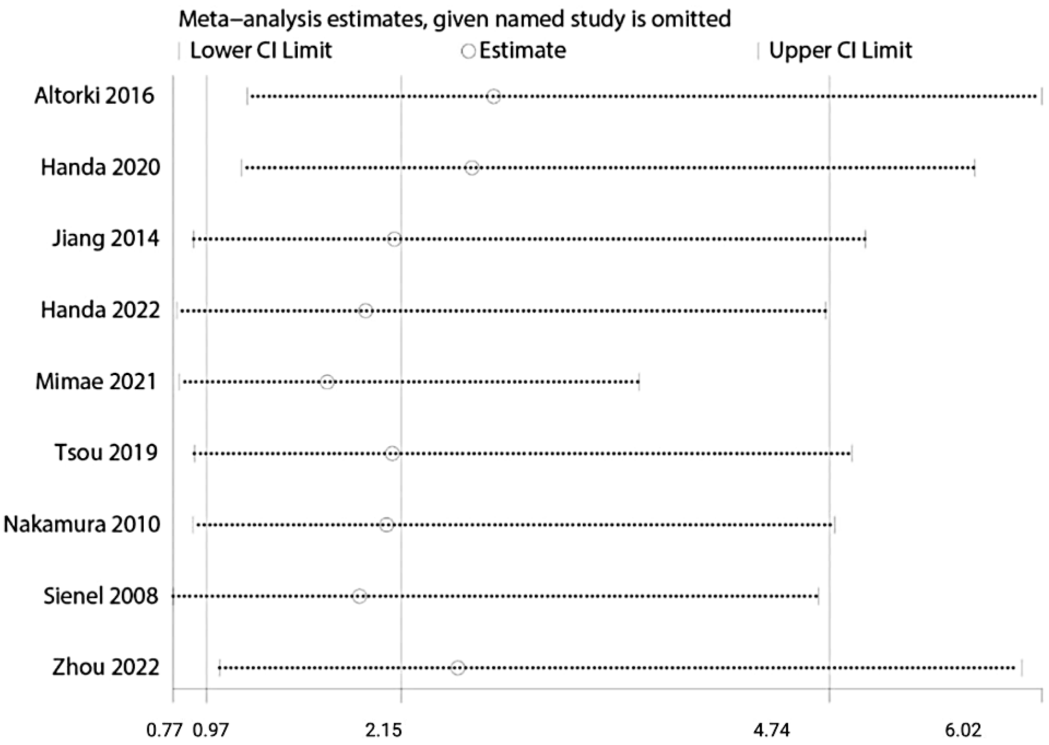

Figure S7. Sensitivity analysis of metastasis rate. CI, confidence interval.

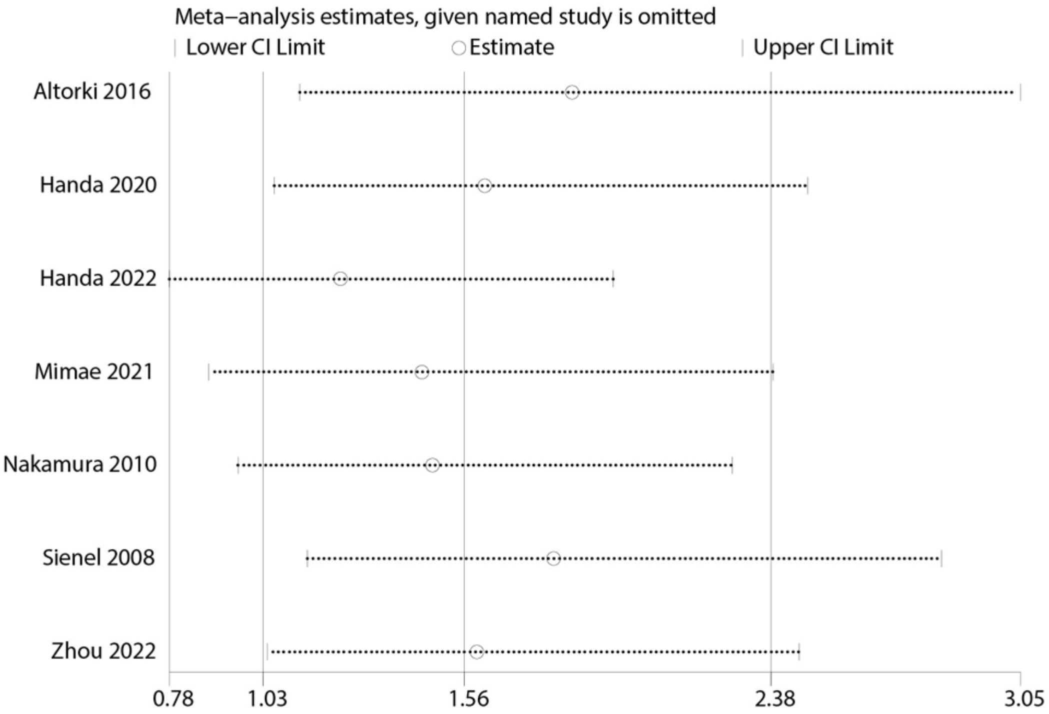

Figure S8. Forest plot of HR of 5-year overall survival in data sources subgroups. HR, hazard ratio; CI, confidence interval.

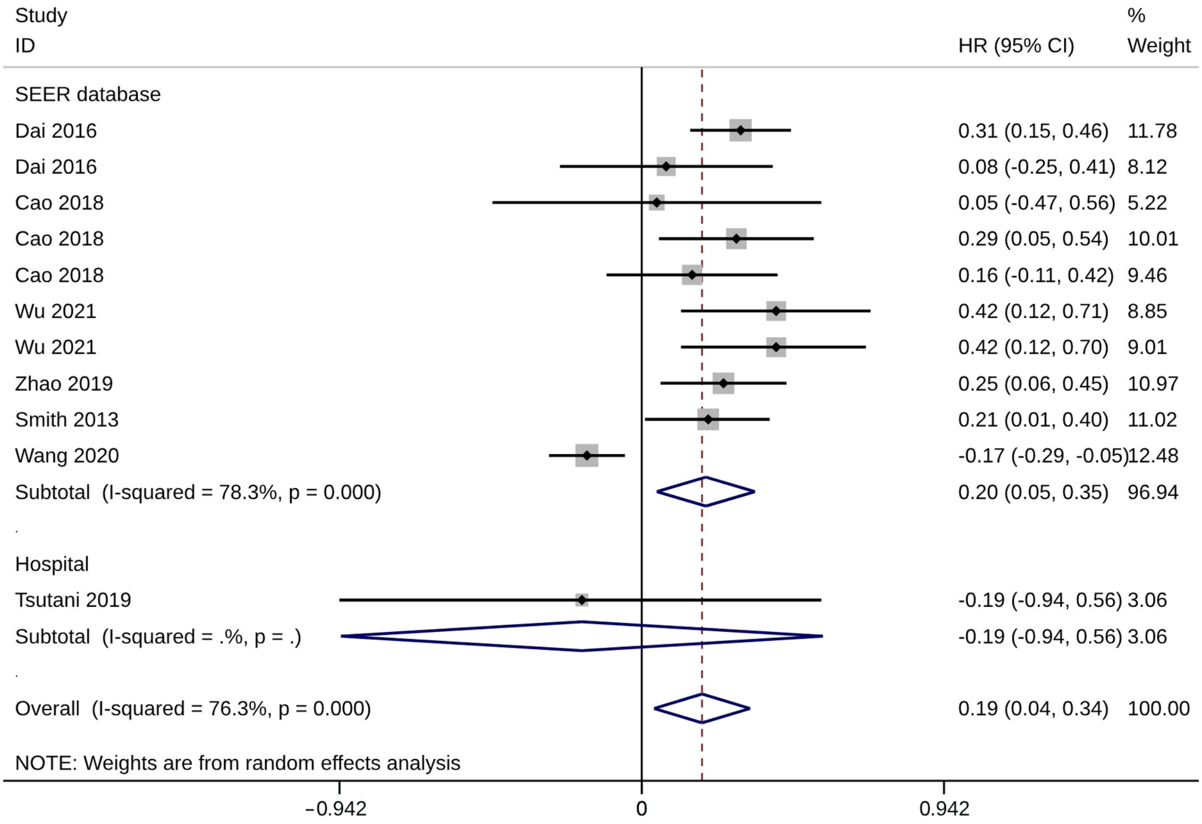

Figure S9. Forest plot of complication in data sources subgroups. OR, odds ratio; CI, confidence interval.

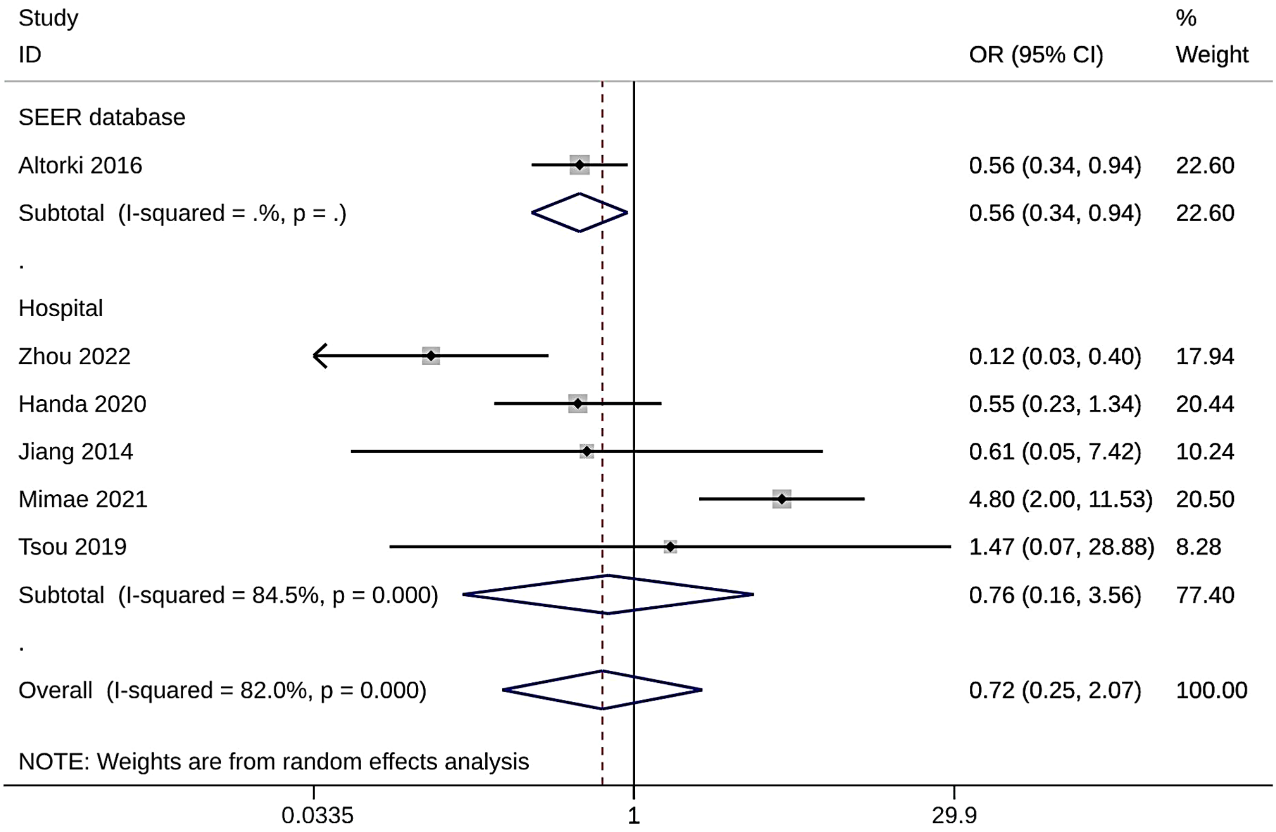

Figure S10. Forest plot of mortality in data sources subgroups. CI, confidence interval; RD, risk difference.

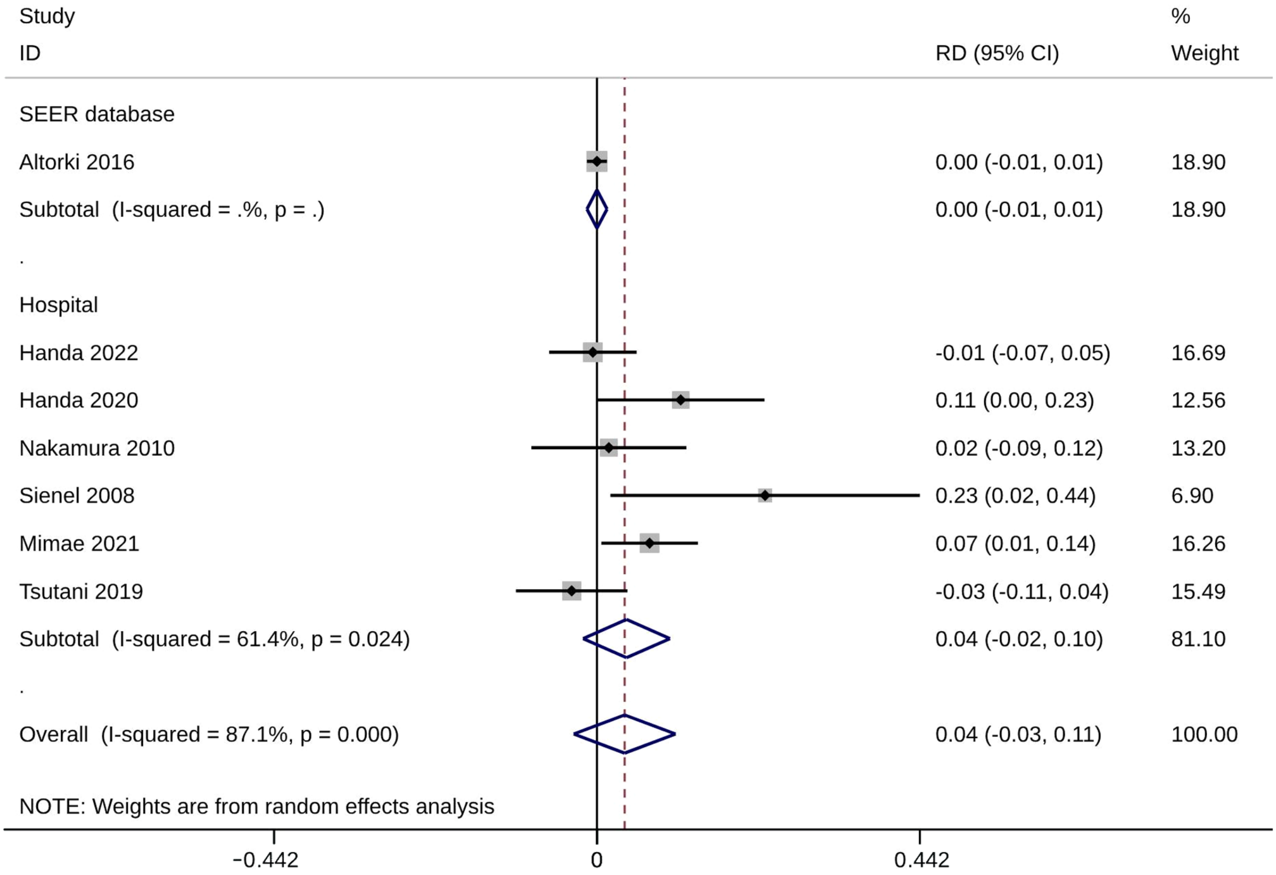

Figure S11. Forest plot of numbers of 5-year overall survival in data sources subgroups. OR, odds ratio; CI, confidence interval.

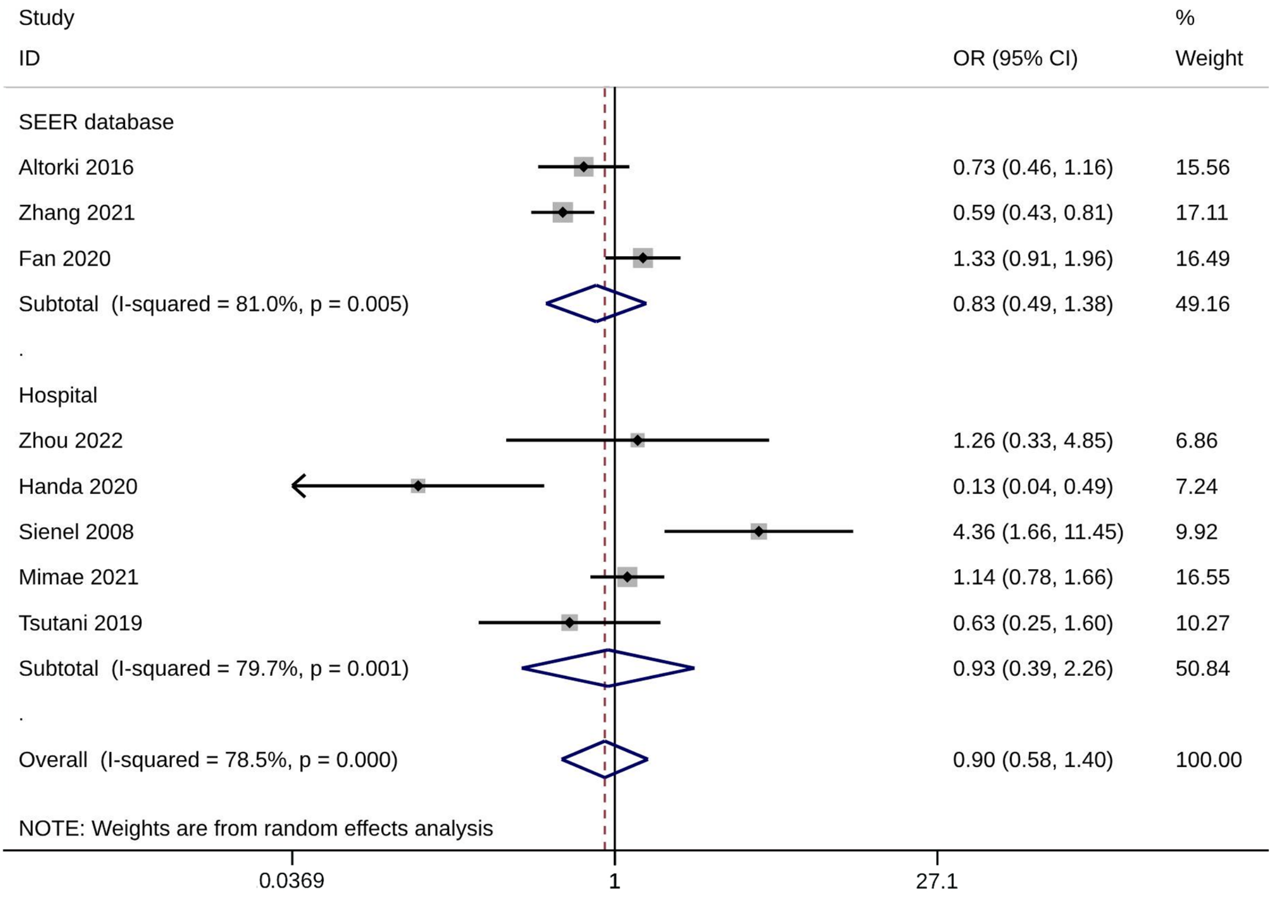

Figure S12. Forest plot of recurrence rate in data sources subgroups. OR, odds ratio; CI, confidence interval.

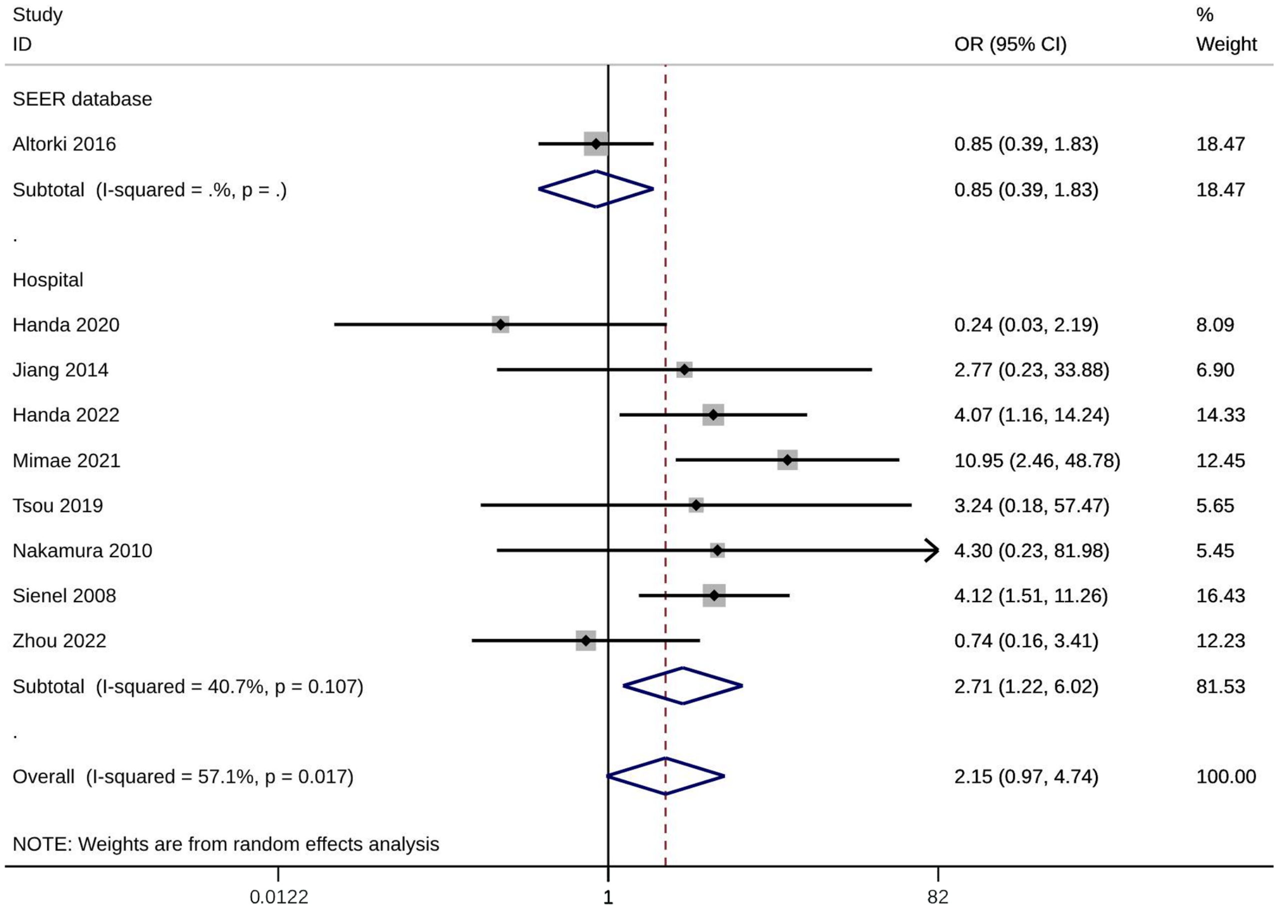

Figure S13. Forest plot of metastasis rate in data sources subgroups. OR, odds ratio; CI, confidence interval.

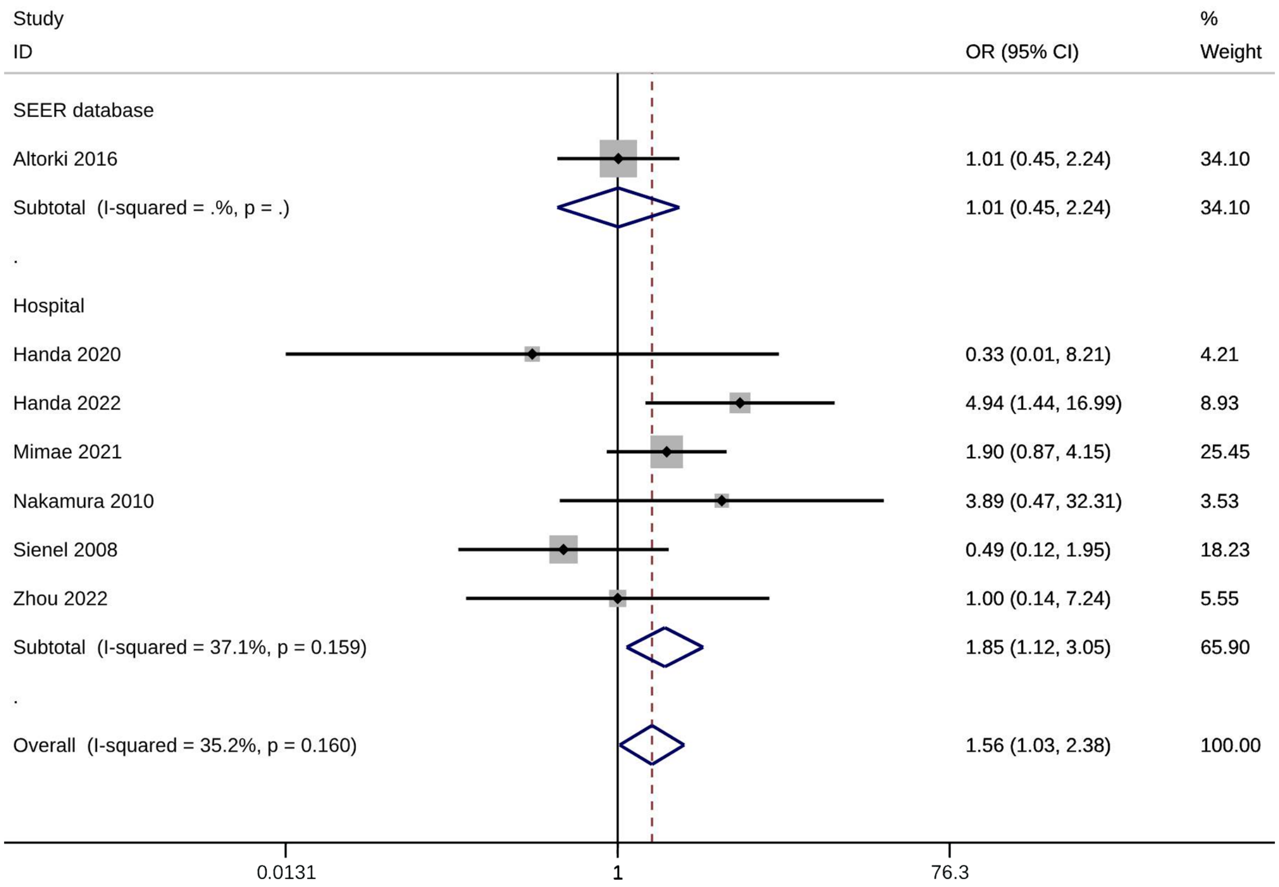

Figure S14. Forest plot of HR of 5-year overall survival in tumour size subgroups. HR, hazard ratio; CI, confidence interval.

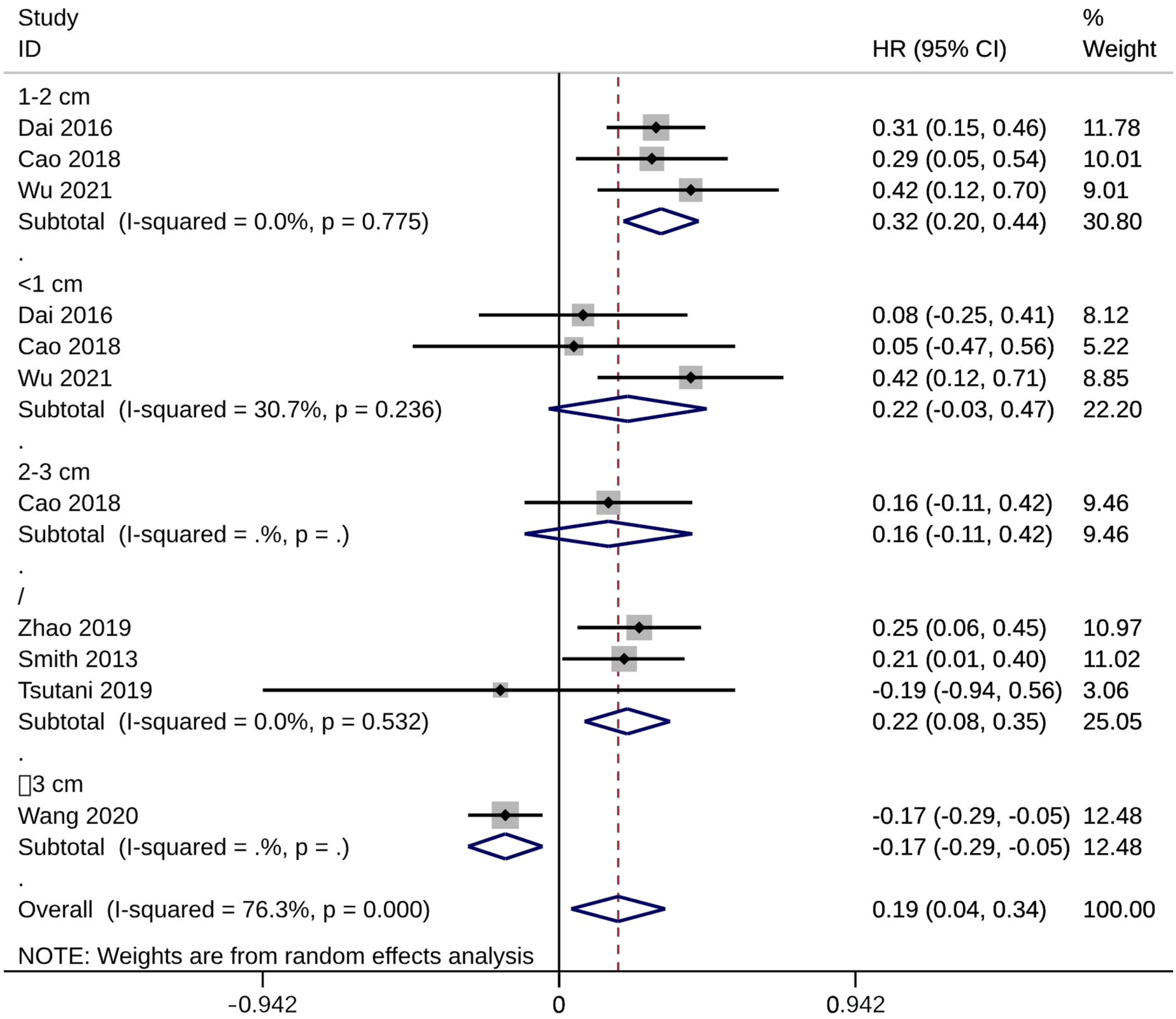

Figure S15. Forest plot of lung cancer-specific survival in tumour size subgroups. HR, hazard ratio; CI, confidence interval.

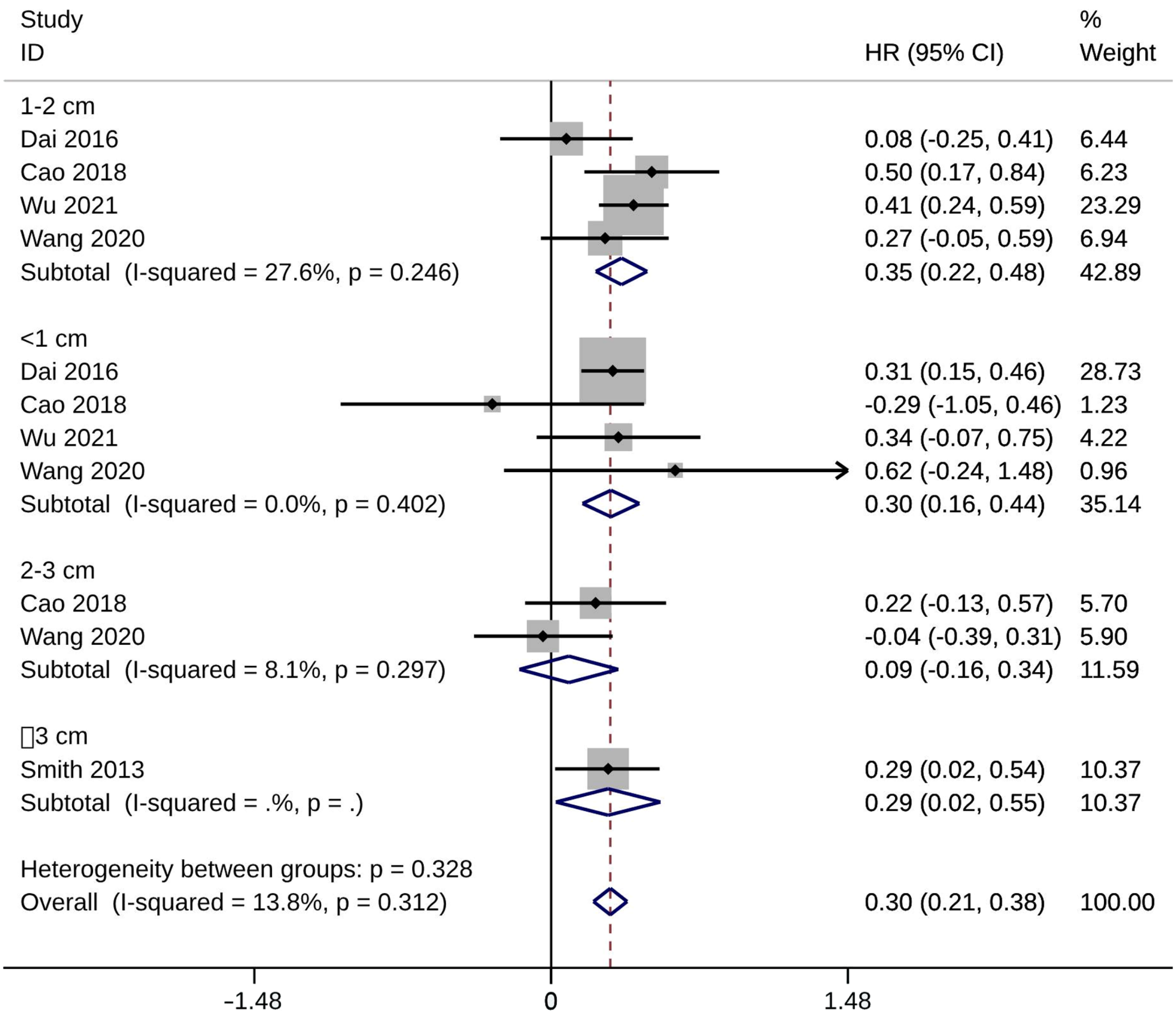

Figure S16. Funnel plot of numbers of 5-year overall survival. OR, odds ratio.

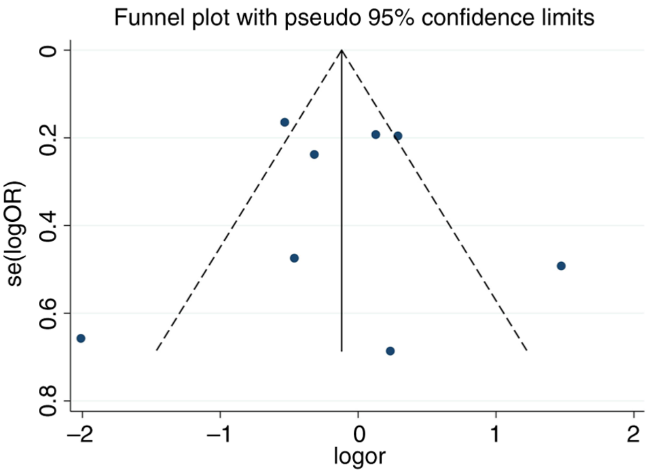

Figure S17. Funnel plot of HR of 5-year overall survival. HR, hazard ratio.

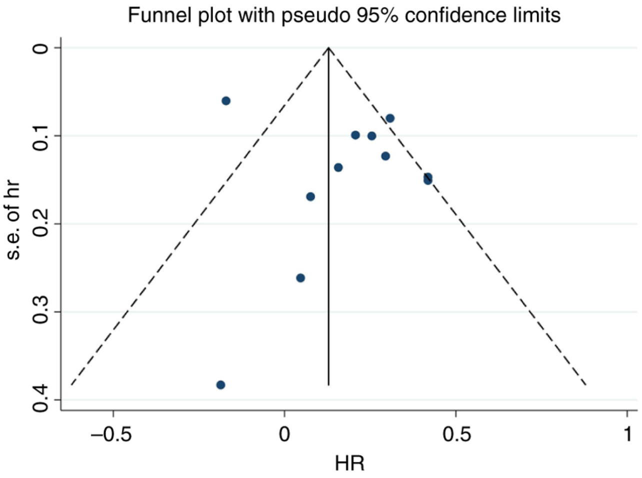

Figure S18. Funnel plot of numbers of lung cancer-specific survival. HR, hazard ratio.

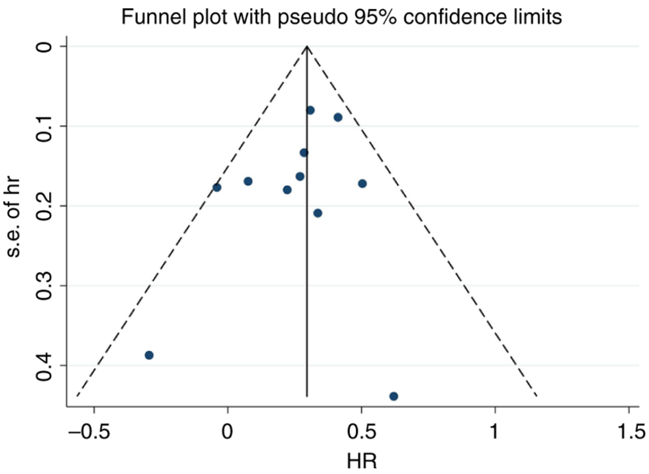

Figure S19. Funnel plot of numbers of mortality. RD, risk difference.

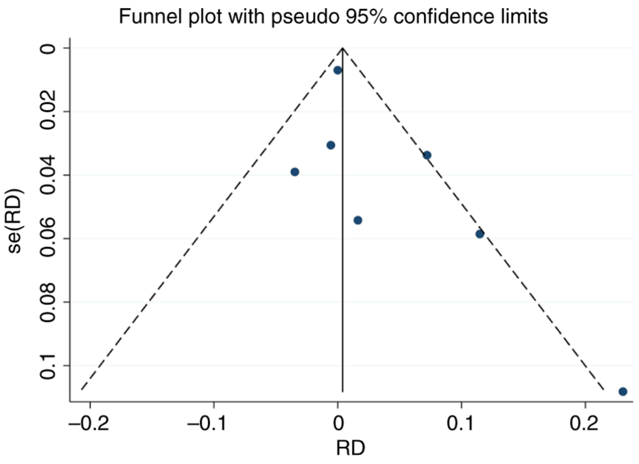

Figure S20. Funnel plot of numbers of complications. OR, odds ratio.

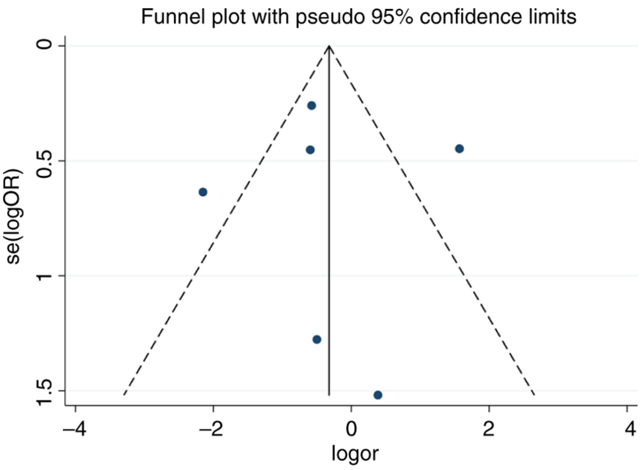

Figure S21. Funnel plot of numbers of recurrence rate. OR, odds ratio.

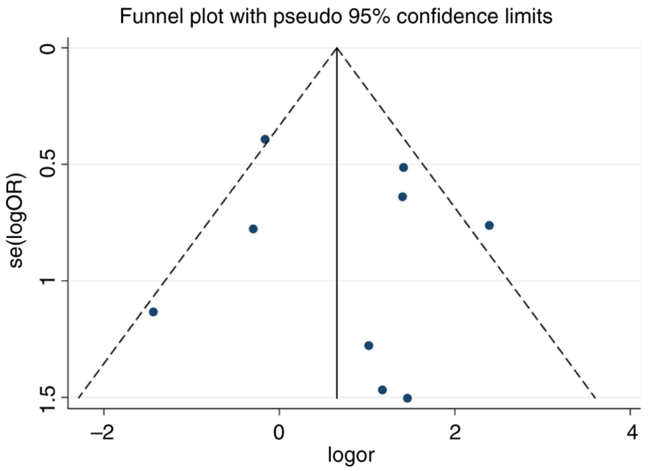

Figure S22. Funnel plot of numbers of metastasis rate. OR, odds ratio.

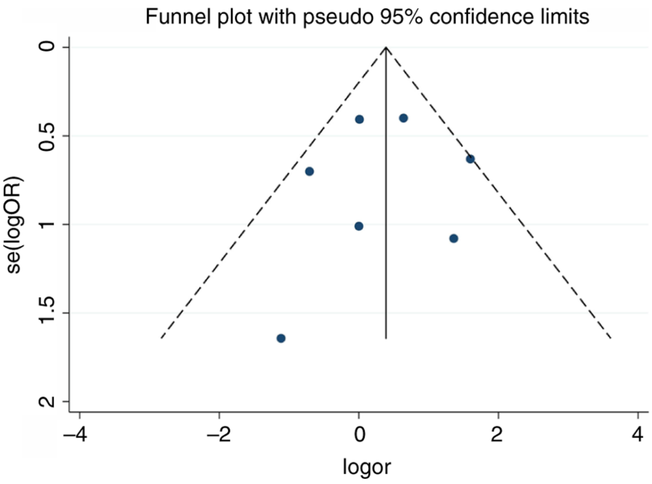

Supplement: Supporting Data [file Supplementary_Data1.pdf]
