# Supplementary material for: Effectiveness and safety of segmentectomy vs. wedge resection for the treatment of patients with operable non‑small cell lung cancer: A meta‑analysis and systematic review
Source: Oncol Lett. 2024 May 24;28(1):336. doi: 10.3892/ol.2024.14469 (PMC11153982; doi:10.3892/ol.2024.14469)
Supplement: Supporting Data [file Supplementary_Data2.pdf]

Table SI. Search strategy.

A, Search strategy of PubMed.		
No.	Search Details	Results
#8	(#1 OR #3) AND (#2 OR #4) AND #5 AND #6 Filters: Humans	296
#7	(#1 OR #3) AND (#2 OR #4) AND #5 AND #6	347
#6	((((segmental excision) OR (segmental exeresis)) OR (segmental resection)) OR (segmental surgical resection)) OR (segmentectomy)	38,929
#5	(((triangular excision) OR (triangular resection)) OR (wedge excision)) OR (wedge resection)	7,615
#4	(((((((((((((((Pneumonectomies) OR (Endoscopic Lung Volume Reduction)) OR (Partial Pneumonectomy)) OR (Partial Pneumonectomies)) OR (Bronchoscopic Lung Volume Reduction)) OR (Lung Volume Reduction)) OR (Lung Volume Reduction Surgery)) OR (limited resection)) OR (sublobar resection)) OR (lung resection)) OR (pneumectomy)) OR	112,916

	(pneumonic resection)) OR (pneumoresection)) OR (pulmonary resection)) OR (pulmonectomy)) OR (resected lung)) OR (pneumonectomy)	
#3	((((((((((((((((((((Non-Small-Cell Lung Carcinomas) OR (Non-Small-Cell Lung Carcinoma)) OR (Non Small Cell Lung Carcinoma)) OR (Non-Small Cell Lung Carcinoma)) OR (Non-Small Cell Lung Cancer)) OR (Nonsmall Cell Lung Cancer)) OR (bronchial non small cell cancer)) OR (bronchial non small cell carcinoma)) OR (lung non small cell cancer)) OR (lung non small cell carcinoma)) OR (non oat cell lung cancer)) OR (non small cell bronchial cancer)) OR (non small cell pulmonary cancer)) OR (non small cell pulmonary carcinoma)) OR (non squamous NSCLC)) OR (non-oat cell lung cancer)) OR (nonsmall cell carcinoma of the lung)) OR (nonsmall cell lung carcinoma)) OR (pulmonary	104,682

	non small cell cancer)) OR (pulmonary non small cell carcinoma)) OR (non small cell lung cancer)	
#2	"Pneumonectomy"[Mesh]	28,986
#1	"Carcinoma, Non-Small-Cell Lung"[Mesh]	70,423
B, Search strategy of EMBASE.		
No.	Search details	Results
#9	(#1 OR #2) AND (#3 OR #4) AND (#5 OR #6) AND (#7 OR #8)	852
#8	'segmental excision':ab,ti,kw OR 'segmental exeresis':ab,ti,kw OR 'segmental resection':ab,ti,kw OR 'segmental surgical resection':ab,ti,kw OR 'segmentectomy':ab,ti,kw	9833
#7	'segmentectomy'/exp	6184
#6	'triangular excision':ab,ti,kw OR 'triangular resection':ab,ti,kw OR 'wedge excision':ab,ti,kw OR 'wedge resection':ab,ti,kw	7897
#5	'wedge resection'/exp	9001
#4	'pneumonectomies':ab,ti,kw OR 'endoscopic lung volume reduction':ab,ti,kw OR 'partial pneumonectomy':ab,ti,kw OR 'partial	31231

	pneumonectomies':ab,ti,kw OR 'bronchoscopic lung volume reduction':ab,ti,kw OR 'lung volume reduction':ab,ti,kw OR 'lung volume reduction surgery':ab,ti,kw OR 'limited resection':ab,ti,kw OR 'sublobar resection':ab,ti,kw OR 'lung resection':ab,ti,kw OR 'pneumectomy':ab,ti,kw OR 'pneumonic resection':ab,ti,kw OR 'pneumoresection':ab,ti,kw OR 'pulmonary resection':ab,ti,kw OR 'pulmonectomy':ab,ti,kw OR 'resected lung':ab,ti,kw OR 'pneumonectomy':ab,ti,kw	
#3	'pneumonectomy'/exp	51174
#2	'non-small-cell lung carcinomas':ab,ti,kw OR 'non-small-cell lung carcinoma':ab,ti,kw OR 'non small cell lung carcinoma':ab,ti,kw OR 'non-small cell lung carcinoma':ab,ti,kw OR 'non-small cell lung cancer':ab,ti,kw OR 'nonsmall cell lung cancer':ab,ti,kw OR 'bronchial non small cell	132518

	cancer':ab,ti,kw OR 'bronchial non small cell carcinoma':ab,ti,kw OR 'lung non small cell cancer':ab,ti,kw OR 'lung non small cell carcinoma':ab,ti,kw OR 'non oat cell lung cancer':ab,ti,kw OR 'non small cell bronchial cancer':ab,ti,kw OR 'non small cell pulmonary cancer':ab,ti,kw OR 'non small cell pulmonary carcinoma':ab,ti,kw OR 'non squamous nsclc':ab,ti,kw OR 'non-oat cell lung cancer':ab,ti,kw OR 'nonsmall cell carcinoma of the lung':ab,ti,kw OR 'nonsmall cell lung carcinoma':ab,ti,kw OR 'pulmonary non small cell cancer':ab,ti,kw OR 'pulmonary non small cell carcinoma':ab,ti,kw OR 'non small cell lung cancer':ab,ti,kw	
#1	'non small cell lung cancer'/exp	210775
C, Search strategy of Cochrane Library.		
No.	Search details	Results

#1	MeSH descriptor: [Carcinoma, Non-Small-Cell Lung] explode all trees	5777
#2	MeSH descriptor: [Pneumonectomy] explode all trees	933
#3	(Non-Small-Cell Lung Carcinomas):ab,ti,kw OR (Non-Small-Cell Lung Carcinoma):ab,ti,kw OR (Non Small Cell Lung Carcinoma):ab,ti,kw OR (Non-Small Cell Lung Carcinoma):ab,ti,kw OR (Non-Small Cell Lung Cancer):ab,ti,kw OR (Nonsmall Cell Lung Cancer):ab,ti,kw OR (bronchial non small cell cancer):ab,ti,kw OR (bronchial non small cell carcinoma):ab,ti,kw OR (lung non small cell cancer):ab,ti,kw OR (lung non small cell carcinoma):ab,ti,kw OR (non oat cell lung cancer):ab,ti,kw OR (non small cell bronchial cancer):ab,ti,kw OR (non small cell pulmonary cancer):ab,ti,kw OR (non small cell pulmonary carcinoma):ab,ti,kw OR (non	16168

	squamous NSCLC):ab,ti,kw OR (non-oat cell lung cancer):ab,ti,kw OR (nonsmall cell carcinoma of the lung):ab,ti,kw OR (nonsmall cell lung carcinoma):ab,ti,kw OR (pulmonary non small cell cancer):ab,ti,kw OR (pulmonary non small cell carcinoma):ab,ti,kw OR (non small cell lung cancer):ab,ti,kw	
#4	(Pneumonectomies):ab,ti,kw OR (Endoscopic Lung Volume Reduction):ab,ti,kw OR (Partial Pneumonectomy):ab,ti,kw OR (Partial Pneumonectomies):ab,ti,kw OR (Bronchoscopic Lung Volume Reduction):ab,ti,kw OR (Lung Volume Reduction):ab,ti,kw OR (Lung Volume Reduction Surgery):ab,ti,kw OR (limited resection):ab,ti,kw OR (sublobar resection):ab,ti,kw OR (lung resection):ab,ti,kw OR (pneumectomy):ab,ti,kw OR (pneumonic resection):ab,ti,kw OR	8490

	(pneumoresection):ab,ti,kw OR (pulmonary resection):ab,ti,kw OR (pulmonectomy):ab,ti,kw OR (resected lung):ab,ti,kw OR (pneumonectomy):ab,ti,kw	
#5	(triangular excision):ab,ti,kw OR (triangular resection):ab,ti,kw OR (wedge excision):ab,ti,kw OR (wedge resection):ab,ti,kw	443
#6	(segmental excision):ab,ti,kw OR (segmental exeresis):ab,ti,kw OR (segmental resection):ab,ti,kw OR (segmental surgical resection):ab,ti,kw OR (segmentectomy):ab,ti,kw	786
#7	(#1 OR #3) AND (#2 OR #4) AND #5 AND #6	48
D, Search strategy of web of science.		
No.	Search details	Results
#1	(((((((((((((((((TS=(Non-Small-Cell Lung Carcinomas) OR TS=(Non-Small-Cell Lung Carcinoma)) OR TS=(Non Small Cell Lung Carcinoma)) OR TS=(Non-	114254

	Small Cell Lung Carcinoma)) OR TS=(Non-Small Cell Lung Cancer)) OR TS=(Nonsmall Cell Lung Cancer)) OR TS=(bronchial non small cell cancer)) OR TS=(bronchial non small cell carcinoma)) OR TS=(lung non small cell cancer)) OR TS=(lung non small cell carcinoma)) OR TS=(non oat cell lung cancer)) OR TS=(non small cell bronchial cancer)) OR TS=(non small cell pulmonary cancer)) OR TS=(non small cell pulmonary carcinoma)) OR TS=(non squamous NSCLC)) OR TS=(non-oat cell lung cancer)) OR TS=(nonsmall cell carcinoma of the lung)) OR TS=(nonsmall cell lung carcinoma)) OR TS=(pulmonary non small cell cancer)) OR TS=(pulmonary non small cell carcinoma)) OR TS=(non small cell lung cancer)	
#2	((((((((((((((TS=(Pneumonectomies) OR	84731

	TS=(Endoscopic Lung Volume Reduction)) OR TS=(Partial Pneumonectomy)) OR TS=(Partial Pneumonectomies)) OR TS=(Bronchoscopic Lung Volume Reduction)) OR TS=(Lung Volume Reduction)) OR TS=(Lung Volume Reduction Surgery)) OR TS=(limited resection)) OR TS=(sublobar resection)) OR TS=(lung resection)) OR TS=(pneumectomy)) OR TS=(pneumonic resection)) OR TS=(pneumoresection)) OR TS=(pulmonary resection)) OR TS=(pulmonectomy)) OR TS=(resected lung)) OR TS=(pneumonectomy)	
#3	((TS=(triangular excision) OR TS=(triangular resection)) OR TS=(wedge excision)) OR TS=(wedge resection)	6087
#4	(((TS=(segmental excision) OR TS=(segmental exeresis)) OR TS=(segmental resection)) OR TS=(segmental surgical	9284

	resection)) OR TS=(segmentectomy)	
#5	#4 AND #3 AND #2 AND #1	324

Table SII. Sensitive analysis of summarized outcomes.

A, Results of sensitive analysis of HR of 5-year overall survival.			
First author(s), year	Estimate	95% confidence interval	(Refs.)
Dai *et al*, 2016	0.171819	0.009506, 0.334133	(26)
Dai *et al*, 2016	0.19771	0.037084, 0.358337	(26)
Cao *et al*, 2018	0.195675	0.039348, 0.352002	(25)
Cao *et al*, 2018	0.175832	0.014259, 0.337405	(25)
Cao *et al*, 2018	0.190882	0.027249, 0.354516	(25)
Wu *et al*, 2021	0.165477	0.010562, 0.320392	(36)
Wu *et al*, 2021	0.165049	0.010218, 0.319881	(36)
Zhao *et al*, 2019	0.179471	0.013695, 0.345248	(39)
Smith *et al*, 2013	0.185181	0.017102, 0.353259	(33)
Tsutani *et al*, 2019	0.19976	0.046917, 0.352603	(35)
Wang *et al*, 2020	0.260133	0.181668, 0.338598	(20)
Combined	0.187832	0.038493, 0.337171	
B, Results of sensitive analysis of numbers of 5-year overall survival.			
First author(s), year	Estimate	95% confidence interval	(Refs.)
Altorki *et al*, 2016	0.934257	0.553457, 1.577062	(24)
Zhang *et al*, 2021	0.984869	0.60246, 1.610012	(19)
Zhou *et al*, 2022	0.879134	0.550762, 1.403288	(37)
Fan *et al*, 2020	0.834962	0.503626, 1.384286	(27)
Handa *et al*, 2021	1.032978	0.68704, 1.553102	(29)
Sienel *et al*, 2008	0.776592	0.521988, 1.155381	(32)
Mimae *et al*, 2021	0.859629	0.502786, 1.469737	(38)
Tsutani *et al*, 2019	0.939894	0.581644, 1.518798	(35)
Combined	0.902396	0.580112, 1.403724	
C, Results of sensitive analysis of lung cancer-specific survival.			
First author(s), year	Estimate	95% confidence interval	(Refs.)
Dai *et al*, 2016	0.310999	0.223999, 0.397999	(26)

Dai *et al*, 2016	0.290877	0.191197, 0.390556	(26)
Cao *et al*, 2018	0.303208	0.218535, 0.38788	(25)
Cao *et al*, 2018	0.282087	0.195186, 0.368987	(25)
Cao *et al*, 2018	0.300302	0.213646, 0.386957	(25)
Smith *et al*, 2013	0.29407	0.208086, 0.380054	(33)
Smith *et al*, 2013	0.260572	0.164496, 0.356649	(33)
Smith *et al*, 2013	0.297096	0.208208, 0.385983	(33)
Wang *et al*, 2020	0.292719	0.208163, 0.377275	(20)
Wang *et al*, 2020	0.297784	0.210555, 0.385014	(20)
Wang *et al*, 2020	0.316952	0.230206, 0.403698	(20)
Combined	0.295859	0.211709, 0.38001	
D, Results of sensitive analysis of mortality.			
First author(s), year	Estimate	95% confidence interval	(Refs.)
Altorki *et al*, 2016	0.040514	-0.01901, 0.100041	(24)
Handa *et al*, 2022	0.054552	-0.05601, 0.165109	(28)
Handa *et al*, 2021	0.024896	-0.04268, 0.092476	(29)
Nakamura *et al*, 2011	0.043245	-0.03806, 0.124548	(31)
Sienel *et al*, 2008	0.021859	-0.03531, 0.079026	(32)
Mimae *et al*, 2021	0.026835	-0.03836, 0.092028	(38)
Tsutani *et al*, 2019	0.054645	-0.033, 0.142292	(35)
Combined	0.037898	-0.03186, 0.107654	
E, Results of sensitive analysis of complication.			
First author(s), year	Estimate	95% confidence interval	(Refs.)
Altorki *et al*, 2016	0.760566	0.162443, 3.561	(24)
Zhou *et al*, 2022	1.062857	0.365828, 3.087965	(37)
Handa *et al*, 2021	0.761435	0.189179, 3.064727	(29)
Jiang *et al*, 2014	0.728744	0.228449, 2.32467	(30)
Mimae *et al*, 2021	0.439278	0.233141, 0.827676	(38)
Tsou *et al*, 2020	0.668468	0.21437, 2.084482	(34)
Combined	0.715216	0.247679, 2.065307	

F, Results of sensitive analysis of recurrence rate.			
First author(s), year	Estimate	95% confidence interval	(Refs.)
Altorki *et al,* 2016	2.708151	1.217736, 6.022723	(24)
Handa *et al*, 2021	2.579884	1.185035, 5.616544	(29)
Jiang *et al*, 2014	2.108956	0.897895, 4.95347	(30)
Handa *et al*, 2022	1.935014	0.793929, 4.71614	(28)
Mimae *et al*, 2021	1.702907	0.808755, 3.585623	(38)
Tsou *et al*, 2020	2.096145	0.901638, 4.873154	(34)
Nakamura *et al*, 2011	2.062792	0.891926, 4.7707	(31)
Sienel *et al*, 2008	1.899072	0.771966, 4.671809	(32)
Zhou *et al*, 2022	2.49385	1.05355, 5.903177	(37)
Combined	2.148814	0.974348, 4.738965	
G, Results of sensitive analysis of metastasis rate.			
First author(s), year	Estimate	95% confidence interval	(Refs.)
Altorki *et al,* 2016	1.851857	1.12379, 3.051615	(24)
Handa *et al*, 2021	1.618709	1.055496, 2.482453	(29)
Handa *et al*, 2022	1.233255	0.775289, 1.961743	(28)
Mimae *et al,* 2021	1.45099	0.881055, 2.389603	(38)
Nakamura *et al,* 2011	1.479096	0.959122, 2.280964	(31)
Sienel *et al*, 2008	1.803158	1.144617, 2.840582	(32)
Zhou *et al*, 2022	1.597485	1.037354, 2.460062	(37)
Combined	1.564322	1.026394, 2.384178	

Table SIII. Publication bias of summarized outcomes.

Outcomes	Publication bias	
	Begg (P-value)	Egger (P-value)
Overall survival	0.436	0.257
Lung cancer-specific survival	0.436	0.263
Mortality	0.072	0.145
Complication	0.707	0.530
Recurrence rate	0.917	0.655
Metastasis rate	1.000	0.827

NA, not available.
